# Transforming and comparing data between standard SQUID and OPM-MEG systems

**DOI:** 10.1371/journal.pone.0262669

**Published:** 2022-01-19

**Authors:** Urban Marhl, Anna Jodko-Władzińska, Rüdiger Brühl, Tilmann Sander, Vojko Jazbinšek

**Affiliations:** 1 Institute of Mathematics, Physics and Mechanics, Ljubljana, Slovenia; 2 Faculty of Natural Sciences and Mathematics, University of Maribor, Maribor, Slovenia; 3 Warsaw University of Technology, Warsaw, Poland; 4 Physikalisch-Technische Bundesanstalt, Berlin, Germany; Cook Children\’s Health Care System: Cook Children’s Medical Center, UNITED STATES

## Abstract

Optically pumped magnetometers (OPMs) have recently become so sensitive that they are suitable for use in magnetoencephalography (MEG). These sensors solve operational problems of the current standard MEG, where superconducting quantum interference device (SQUID) gradiometers and magnetometers are being used. The main advantage of OPMs is that they do not require cryogenics for cooling. Therefore, they can be placed closer to the scalp and are much easier to use. Here, we measured auditory evoked fields (AEFs) with both SQUID- and OPM-based MEG systems for a group of subjects to better understand the usage of a limited sensor count OPM-MEG. We present a theoretical framework that transforms the within subject data and equivalent simulation data from one MEG system to the other. This approach works on the principle of solving the inverse problem with one system, and then using the forward model to calculate the magnetic fields expected for the other system. For the source reconstruction, we used a minimum norm estimate (MNE) of the current distribution. Two different volume conductor models were compared: the homogeneous conducting sphere and the three-shell model of the head. The transformation results are characterized by a relative error and cross-correlation between the measured and the estimated magnetic field maps of the AEFs. The results for both models are encouraging. Since some commercial OPMs measure multiple components of the magnetic field simultaneously, we additionally analyzed the effect of tangential field components. Overall, our dual-axis OPM-MEG with 15 sensors yields similar information to a 62-channel SQUID-MEG with its field of view restricted to the right hemisphere.

## Introduction

Magnetoencephalography (MEG) is a neuroimaging technique that measures the magnetic field in the vicinity of the head [1, 2]. It is widely used in cognitive neuroscience, but only rarely in clinical practice as its difficult and expensive operation limits its incorporation into clinical routine. Standard MEG systems use superconducting quantum interference devices (SQUIDs), which can be configured as a magnetometer/gradiometer to measure very small magnetic fields suitable to detect brain or heart activity [3, 4]. SQUIDs consist of superconductor rings separated by Josephson junctions; therefore, they need a complicated cooling system with cryogenics to operate. Following miniaturization [5, 6], a new type of magnetometer, the optically-pumped magnetometer (OPM), was introduced to MEG [7, 8]. OPMs work on a completely different physical principle compared to SQUIDs. OPMs measure transmission of monochromatic light through a cell of hot vapors of alkali metals, and the transmission depends on the strength of the magnetic field [9].

At current prices of OPMs (several thousand dollars per sensor), a full-head coverage OPM-MEG employing 50 and more sensors is a high initial investment in the range of SQUID-MEG (the cost of magnetic shielding is not taken into consideration therein). Therefore, current OPM-MEG setups often do not aim for full head coverage since the lack of available sensors can be offset by the flexibility in placing the OPMs around the scalp. For this reason, exploring the applicability of an OPM-MEG setup with a limited sensor count is of great importance, and its usefulness was demonstrated in [10].

Many studies evaluate the performance of OPM-MEG and SQUID-MEG by comparing the reconstruction results in the source space [10–13]. Instead, we compare the performance of two systems in the sensor space. Comparing the transformed and the measured data in both sensor spaces has many advantages. For example, we can determine which system measures more spatial information. If the transformation error is greater when transforming data from the first to the second system than when transforming data from the second to the first system, then the layout of the second system most likely measures more spatial information.

The geometry and the underlying physical sensor principle of OPM-MEG and SQUID-MEG differ greatly. To be able to compare the results of these two types of devices in sensor space, a suitable transformation needs to be found. In this work, we use a transformation approach that was originally developed to compare and transform results from different SQUID layouts for both MEG and magnetocardiography [14–20]. This approach works on calculating the source reconstruction (solving the inverse problem) with one system, and then using this result to calculate the magnetic field on the other system using the forward model. The most suitable source reconstruction method for this is the minimum norm estimate (MNE) method [21], but other methods could also be used. This transformation technique could also be used to plan measurements with a low count OPM-MEG system if a whole head SQUID-MEG system is available and no prior physiological information is known.

The level of noise in data affects the transformation error. This was already shown in a study where they used phantom data [19]. They showed that the method of transforming MEG recordings to a standard sensor layout performs very well, also for large distortions and high levels of noise. To explore the effects of noise on the transformation error and have as much control as possible, we apply the transformations to simulated data closely matching the experimental data. In the simulation, the magnetic fields are calculated using a forward model incorporating anatomical and functional prior knowledge [22–25].

As a “model” brain response we measured AEFs [26, 27] for a group of subjects using both a full-head coverage SQUID- and a limited sensor count OPM-MEG. AEFs are appropriate for this study since they are stable and highly reproducible both from a temporal and structural point of view. Although the focus was on the case of AEFs, it is expected that the methods in this study can be applied to other brain sources. The OPM-MEG system consists of 15 sensors, each measuring 2 orthogonal components simultaneously (radial and tangential relative to the head surface); the SQUID-MEG system is a 125-channel array of axial gradiometers. For OPM-MEG, the 3D-printed sensor holder approach demonstrated in [10] was used.

To transform the SQUID data to the OPM layout and vice versa, two MNE implementations are used, one in the Python software package MNE-Python [28], where a realistic conductor model (boundary element method—BEM) [29] is applied. The other is our own Python implementation, where a simplified homogeneous spherical volume conductor is applied [30]. Both methods were compared on measured and simulated AEFs. Comparison between the measured, simulated, and transformed AEF magnetic field maps (MFMs) is evaluated by the relative error (RE) and the correlation coefficient (CC) [31, 32].

The paper is organized as follows: Our experimental OPM and SQUID-MEG setups are introduced, and then the measurements to obtain AEFs are described. The next section explains the theoretical methods that are used to transform the within-subject data from one MEG system to the other. These methods are applied to the experimental and the simulated data, followed by a discussion of the results and, finally, conclusions are made.

## Materials and methods

### Measuring AEF MEG signals

We measured AEFs with both SQUID- and OPM-MEG systems on 8 healthy normal subjects. Participants were independent members of staff and outside participants recruited through adverts, the age group was from 20 to 60. Non-removable metallic objects outside or inside the body were an exclusion criterion except for normal dental work with non-magnetic materials. All MEG measurements were performed inside a conventional magnetically shielded room (MSR) at the Physikalisch-Technische Bundesanstalt (PTB) in Berlin (AK3b, VAC, Hanau, Germany). The study was performed in accordance with the Declaration of Helsinki for research on human subjects, and approved by the Institutional Review Board (IRB) of PTB (PTB2019–1, MEG with OPMs). Written informed consent was given by the participants.

**Fig 1:**
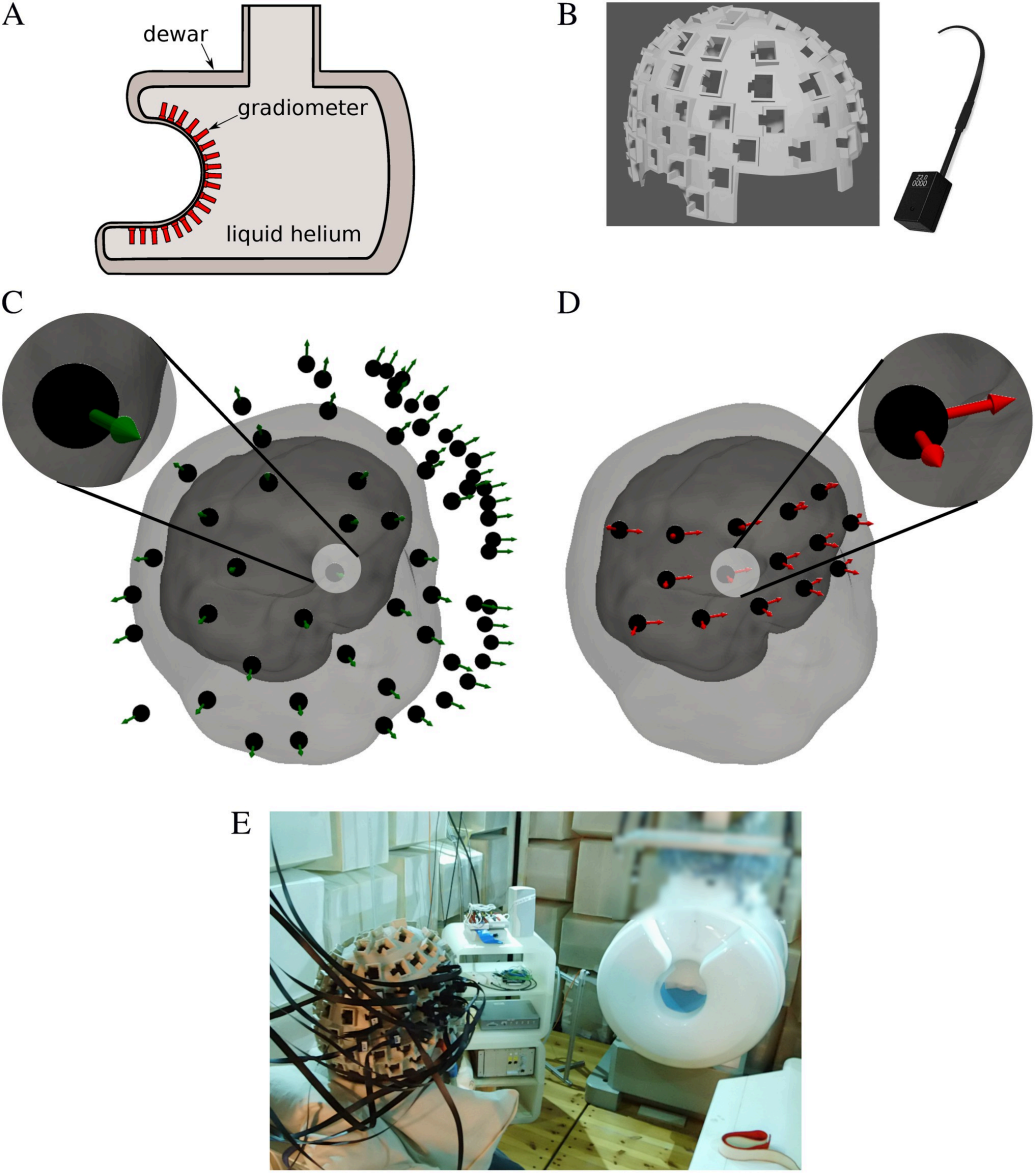
Experimental setup of the SQUID and OPM-MEG systems. (A) Schematic representation of a SQUID-MEG system with a gradiometer array inside a liquid helium Dewar. (B) Dual-axis magnetometers produced by the company QuSpin, and an exemplary CAD model of one sensor holder. (C) Display of the subject’s head surface segmented from an MRI within the SQUID-MEG, and (D) OPM-MEG system. The SQUID-MEG system sensors cover the whole head; for this work we used only one hemisphere, the same as the OPM-MEG system. (E) Photo of one subject wearing the OPM-MEG sensor holder with OPMs placed on the right hemisphere. In the background, the SQUID-MEG system is visible.

The SQUID system consists of 125 axial first-order gradiometers. It was produced by the company Yokogawa [33], and is shown in Fig 1(A) and 1(E). To obtain the anatomical magnetic resonance images (MRI) of an individual subject as input for the OPM-MEG sensor holder, a clinical 3-Tesla scanner (Verio, Siemens Healthcare, Munich, Germany) was used, also located at PTB. A T1 weighted 3D image of the subject’s head was obtained with the MPRAGE (Magnetization Prepared—RApid Gradient Echo) sequence set to 1 mm isotropic resolution. Surface points describing the interface between the air and tissue were extracted and converted to a layer 3 mm thick [34]. To this layer, the slots holding the OPMs were added by computer-aided design (CAD) software. The complete holder was 3D-printed using a combination of standard hard and flexible (soft) polylactic acid (PLA) filament. The CAD model and the real sensor holder fitted with 15 dual-axis OPM sensors (QZFM Gen-2, made by the company QuSpin) [35] are shown in Fig 1(B) and 1(E). The fin between the eye cuttings in Fig 1B ends at the height of the nasion, and is used for aligning the sensor holder on the subject’s head. OPM-MEG sensors were placed on the right hemisphere in a grid around the position C4 on the standard 10–20 EEG system, which is the area known to exhibit large AEFs.

One axis of the OPM sensor is the radial direction of the subject’s head surface, and the other is the tangential direction along the lines of latitude; both directions were recorded simultaneously. The sensor measurement error is specified at 5% by the company, and crosstalk is negligible at sensor distances above 2 cm [35], which is fulfilled by our sensor holder. For a dual-axis sensor, the orthogonality of the axes is of importance particularly if the data are used to estimate source configurations. Therefore, the intrinsic orthogonality between the axes was determined experimentally as described in the (S1 Appendix). An orthogonality error of less than 3% was found. Similar measurement errors are achieved in SQUID systems, and the orthogonality error is not relevant or has not been reported for them.

An experimental setup and sensor layout of both MEG systems are shown in Fig 1. The AEFs were the result of 500 sound stimulations in both ears by a 1 kHz tone lasting for 500 ms, and with an interstimulus interval of 1.2 s. Before the measurement, the subjects indicated comfortable sound levels, and individual sound pressure levels were between 85 and 102 dB. An Etymotic ER-30 tubular insert earphone (www.etymotic.com) was used for stimulation. It consists of an electro-dynamic transducer attached to a 5 m long low-acoustic-damping flexible tube with a foam insertion earpiece. The transducer is outside of the MSR, and the air column along the tube delivers the pressure wave to the ear canal. The stimulation is therefore identical to an in-ear headphone as used for normal listening. The long tube leads to a frequency cut-off at 1.5 kHz, but at 1 kHz the stimulation is not affected by the cut-off. The insert earphone is electrically driven by an AC-coupled audio amplifier, and the electrical signal delivered to the earphone is recorded in an electrical channel synchronous with the MEG signals for offline inspection.

Subjects move from the OPM-MEG setup to the SQUID-MEG setup, and need to remove the insert earphone and re-insert it after placement in the SQUID-MEG. This re-insertion might lead to amplitude differences between the two measurements, and this might explain some variation in the brain current results obtained within a single subject. For our group results, it is assumed that this variation is uncorrelated to other effects.

To determine the head position inside the SQUID-MEG system, 5 marker coils are attached to the subject’s head, and are energized and localized. For each subject, we also measured 20 head shape points and fiducials in relation to the 5 detection coils with the CMS-HS motion analysis system made by the company Zebris Medical GmbH. When measuring with the OPM system, no further co-registration is needed by design since the sensor positions of the individual MRI-derived sensor holder are already given in the head coordinate system.

Our MSR of type Ak3b has two layers of mu-metal and an active compensation [36], which keeps field fluctuations below +-1 nT, as required for linear operation of the OPMs [35].

#### Preprocessing the AEF

Most of the preprocessing was done with the MNE-Python software [37]. We imported the raw measurements that were in a binary system measuring format (extension.con), and converted into Elekta NeuroMag (.fif) format. The sampling frequency for all subjects was 500 Hz. Next, we filtered the raw signal using a finite impulse response (FIR) filter with the window design method implemented in the Python package SciPy [38]. For both (SQUID and OPM) systems, we applied a 4–40 Hz bandpass filter. In SQUID-MEG, a high-pass filter of 0.5–1 Hz is customary, but involuntary movement-related signals in the OPM-MEG data can be dominant up to 3 Hz. These signals are generated by the OPM sensors attached to the head involuntarily moving relative to the static gradients in the MSR. In SQUID-MEG, the sensor array is fixed to the laboratory floor, and only vibrations of the floor can lead to gradient-related noise signals. Since we do not want to study sustained evoked fields [39], a suitable high-pass frequency is 4 Hz, which preserves the M100 response. The low pass frequency was chosen at 40 Hz to remove power line related 50 Hz signals, which are difficult to avoid due to the digital electronics of each OPM sensor. With this low pass filter, faster auditory components such as the M(P)30 are attenuated and were not studied here. From the measured trigger channel, we identified the starting times of the tone onset. With these times, we created 500 epochs, which we then averaged to obtain the evoked AEF response. Using the epochs, we calculated the noise covariance matrix. This matrix is estimated on pre-stimulus periods (from –0.2 s to 0.0 s before the stimulus onset), its diagonal elements are the noise variances of the sensors; we used the function implemented in MNE-Python. During the preprocessing, we identified and excluded the bad/faulty channels by visual inspection of the measured data, e.g., SQUIDs occasionally do not lock, or OPMs do not successfully compensate internally.

In the evoked AEF response, we wanted to find the time of the characteristic M100 response. In order to find it, we calculated the standard deviation (STD) of MFM, presented as an N-dimensional vector of magnetic fields **M** = (*M*_1_, *M*_2_, …, *M*_*N*_), at each time point (*t*):

$$\begin{array}{c} \text{STD}\left(t\right)=\sqrt{\frac{1}{N}\sum_{i=1}^{N}{\left(M_{i}\left(t\right)-\bar{M}\left(t\right)\right)}^{2}}, \end{array}$$ (1)

where *N* is the number of sensors, *M*_*i*_(*t*) is the magnetic field at the i-th sensor averaged over all epochs, and $\bar{M}$ denotes the mean MFM value at time t. The STD is a measure of MFM spatial variability at the sensor locations, and thus estimates the neural activity at a given time. To determine the exact time of the M100 peak, we identified the highest peak of the STD function around the time *t* = 100ms. The average temporal location of M100 for all measurements (OPMs and SQUIDs) was *t* = 107±13ms.

From the processed evoked AEF data, we determined for each subject and MEG system (OPM and SQUID) signal-to-noise ratio (SNR), defined as:

$$\begin{array}{c} \text{SNR}=20\;{\text{log}}_{10}\frac{{\text{RMS}}_{\text{signal}}}{{\text{RMS}}_{\text{noise}}}, \end{array}$$ (2)

where the root mean square (RMS) value of noise (RMS_noise_) was calculated on the 0.2 s time interval before the sound stimulation and the RMS value of the signal (RMS_signal_) was calculated for the time interval ±0.02s around the identified M100 peak.

### Simulation of AEF MEG data

To calculate the simulated data, we located a dipole in the right auditory cortex of each subject’s individual anatomy in accordance with the experiment. We determined the auditory cortex area (anterior transverse temporal gyrus) with the FreeSurfer software [34, 40] by using the Destrieux Atlas [41]. The exact location of the dipole was at the center of the parcellation, this is also where we approximately expect the source of the measured M100 response. Dipole strength was 100 nAm. The direction of the dipole was oriented tangentially to a sphere that was fitted to the outer brain surface points. The MFM of the dipole was then calculated with the built-in function of MNE-Python using the individual geometry of each subject. A 3-layer BEM mesh model (outer scalp, inner scalp and outer brain) was constructed using the watershed algorithm implemented in MNE-Python [28, 37] and FreeSurfer. Each BEM layer consists of 2562 vertices and 5120 triangles. For the outer scalp and the brain compartment, the conductivity was set to 0.3 S/m. The skull conductivity is much smaller and was set to the customary 0.006 S/m. The position of the subject’s head inside the SQUID and OPM-MEG system of the simulated data was the same as in the measurements. To better compare the results of the measurements and simulations, we used only the right hemispheric SQUID sensors.

We also studied the influence of noise on the simulated MFM (**S**). We added uncorrelated Gaussian noise (*ξ*_*i*_) to obtain the noisy MFM (**M**) for the *i*-th sensor *M*_*i*_ = *S*_*i*_ + RMS_noise_ *ξ*_*i*_, where the noise level was set by the *RMS*_noise_, and the corresponding SNR was calculated by the Eq (2), where RMS_signal_ represents the RMS value of the MFM(**S**).

### Transformation of data from one MEG system to another

Here we present the basic methods and parameters that were used to transform the SQUID data to the OPM sensor locations and orientations, and vice versa. The key idea is to calculate the source reconstruction, i.e., finding the inverse solution of the measured MFM at one specific time. Using the inverse solution for one system, we calculate the magnetic fields on the other system.

**Fig 2:**
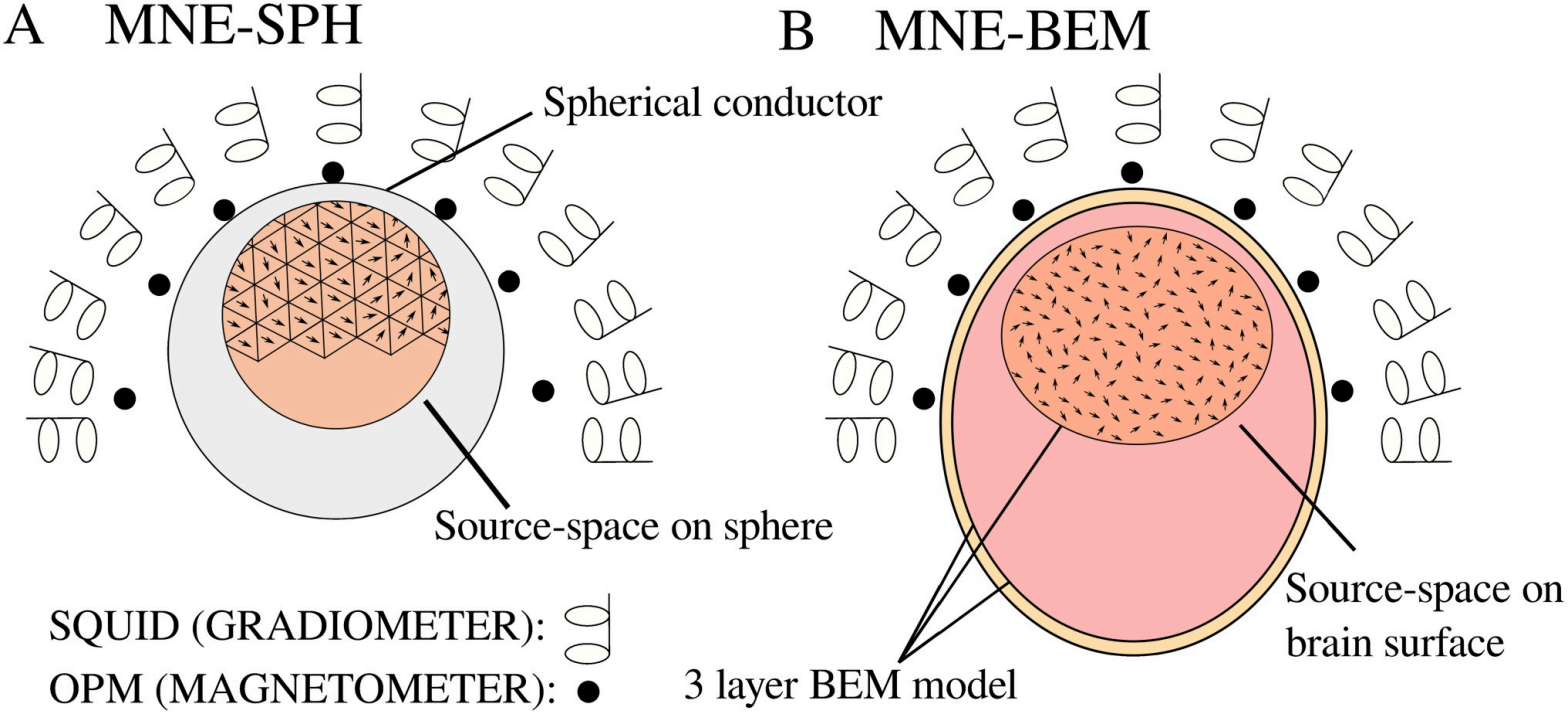
Schematic representation of two different transformation techniques. (A) current source distribution located on the faces of an icosahedron calculated with the MNE method using the homogeneous spherical volume conductor model (MNE-SPH method), and (B) current source distribution on the subject’s reconstructed brain surface, calculated with the MNE method and individual geometry of the head (MNE-BEM method). In the figures, sensors from both MEG systems are drawn; however, we did not use them simultaneously.

For the source reconstruction, we used the MNE algorithm implemented in the MNE-Python software package as well as a simplified implementation of the MNE algorithm. A sketch of each method is shown in Fig 2. When transforming the measured and simulated data, we used only the right half of the SQUID sensors since the OPM sensors measured exclusively in the vicinity of the right auditory cortex. For a more detailed description of these two methods and the forward models, see S2 Appendix.

#### MNE method with a homogeneous spherical volume conductor model and source space distributed on a sphere (MNE-SPH)

This method uses an implementation of the MNE method as described in [30], working on similar principles as the MNE method implemented in MNE-Python (explained below), but with some differences. The sensor model in this implementation uses a point model instead of integration over the sensing area respective volume. For the OPM sensor the center of the gas cell is used, for the SQUID gradiometers the center of each coil is used. The MNE method is generally based on the selection of source space consisting of a large number of current dipoles, and the forward solution, which calculates the magnetic field at the sensor locations from the dipoles in the selected source space. The forward solution in MNE-SPH uses a simplified model of the current dipole source inside a homogeneous spherical volume conductor [30]. A detailed description of the forward model is given in the first paragraph of S2 Appendix. The chosen source space consists of dipoles located on the upper part of a sphere that best fits the outer brain surface. The exact locations are the chosen centers of faces of an upsampled icosahedron with 320 faces and 162 vertices. In our case, we used the top 275 faces. The radius and the center of the icosahedron were chosen individually for each subject depending on the coregistration between the head and the MEG system.

Next, we briefly explain all the major steps of our MNE implementation. A detailed description of this implementation is in the third paragraph of S2 Appendix. First, we calculated the lead fields $\vec{L}$ and a lead field matrix **Γ**, which is a linear operator that calculates the magnetic field from a known source distribution. Since the inverse problem is ill-posed, we have to regularize it [42]. To calculate the inverse, we used a simplified truncated SVD approach [43]. Using the Moore-Penrose inverse of matrix, we calculated **Γ**^−1^ [44]. To calculate the weight **w**, we used the relation **w** = **Γ**^−1^**B**. With the weight **w** and all the lead fields $\vec{L}$, we can calculate the strength of all three components for the *i*-th dipole in the source space:

$$\begin{array}{c} {\vec{P}}_{i}=\sum_{j=1}^{n}w_{j}{\vec{L}}_{j,i}, \end{array}$$ (3)

where *n* is the number of all sensors. Using the solution ${\vec{P}}_{i}$, we can then calculate the magnetic field on another MEG system, but we need to calculate the lead fields ${\vec{L}}^{\prime }$ of the MEG system to which we want to transform. The magnetic field $B^{\prime }=\left(B_{1}^{\prime },B_{2}^{\prime },\ldots ,B_{n}\right)$ is then calculated as:

$$\begin{array}{c} B_{j}^{\prime }=\sum_{i=1}^{m}{\vec{L}}_{j,i}^{\prime }\cdot {\vec{P}}_{i}, \end{array}$$ (4)

where *m* is the number of all elements in the source space.

#### MNE method implemented in MNE-Python using subject’s individual BEM model (MNE-BEM) [28]

This source reconstruction uses a forward model that takes into account the individual geometry for each subject (BEM method). A description of this forward model can be found in the second paragraph of S2 Appendix. The sensor model in this implementation takes into account the real sensor geometry. The source space is surface-based and is distributed evenly over the outer surface of both hemispheres. Each hemisphere has around 4000 vertices. Each vertex represents a current dipole with a fixed orientation. For BEM calculations, the linear collocation method [45] with isolated skull approach [46] is used. A description of MNE, including an example code, is presented in the third paragraph of S2 Appendix. In MNE-Python, the inverse operator is calculated with the forward model and the noise covariance matrix. The noise covariance matrix represents the measure of the noise level in channels, and is used in the inverse solution to define which lead fields add more weight to the solution (less noisy channels have greater power), as well as in the regularization of the solution. As a result, we obtained the source estimate, i.e., the parameters of each current dipole (${\vec{P}}_{i}$) in the source space. Like in the MNE-SPH method, the transformation of data to the other MEG system is done by applying (4) using lead fields (${\vec{L}}^{\prime }$) of the MEG system to which we want to transform.

### Evaluation parameters of measurements and calculations

To compare two MFMs, the measured **M** and the estimated **E**, with the same sensor layout, we used two measures: the relative root mean square error (RE) and the linear correlation coefficient, i.e., Pearson’s CC [47]

$$\begin{array}{c} \text{RE}=\frac{\sqrt{\bar{{\left(E-M\right)}^{2}}}}{\sqrt{\bar{M^{2}}}}, \end{array}$$ (5)

$$\begin{array}{c} \text{CC}=\frac{\sum_{i=1}^{N}\left(M_{i}-\bar{M}\right)\left(E_{i}-\bar{E}\right)}{\sqrt{\sum_{i=1}^{N}{\left(M_{i}-\bar{M}\right)}^{2}}\sqrt{\sum_{i=1}^{N}{\left(E_{i}-\bar{E}\right)}^{2}}}. \end{array}$$ (6)

CC measures linear correlation between two variables, its value can be from –1 to 1. The closer its absolute value to value 1, the more the shapes of MFMs are similar. RE depends on the signal amplitude at each sensor location. The closer the value is to 0, the more similarities can be identified in compared MFMs.

## Results

**Fig 3:**
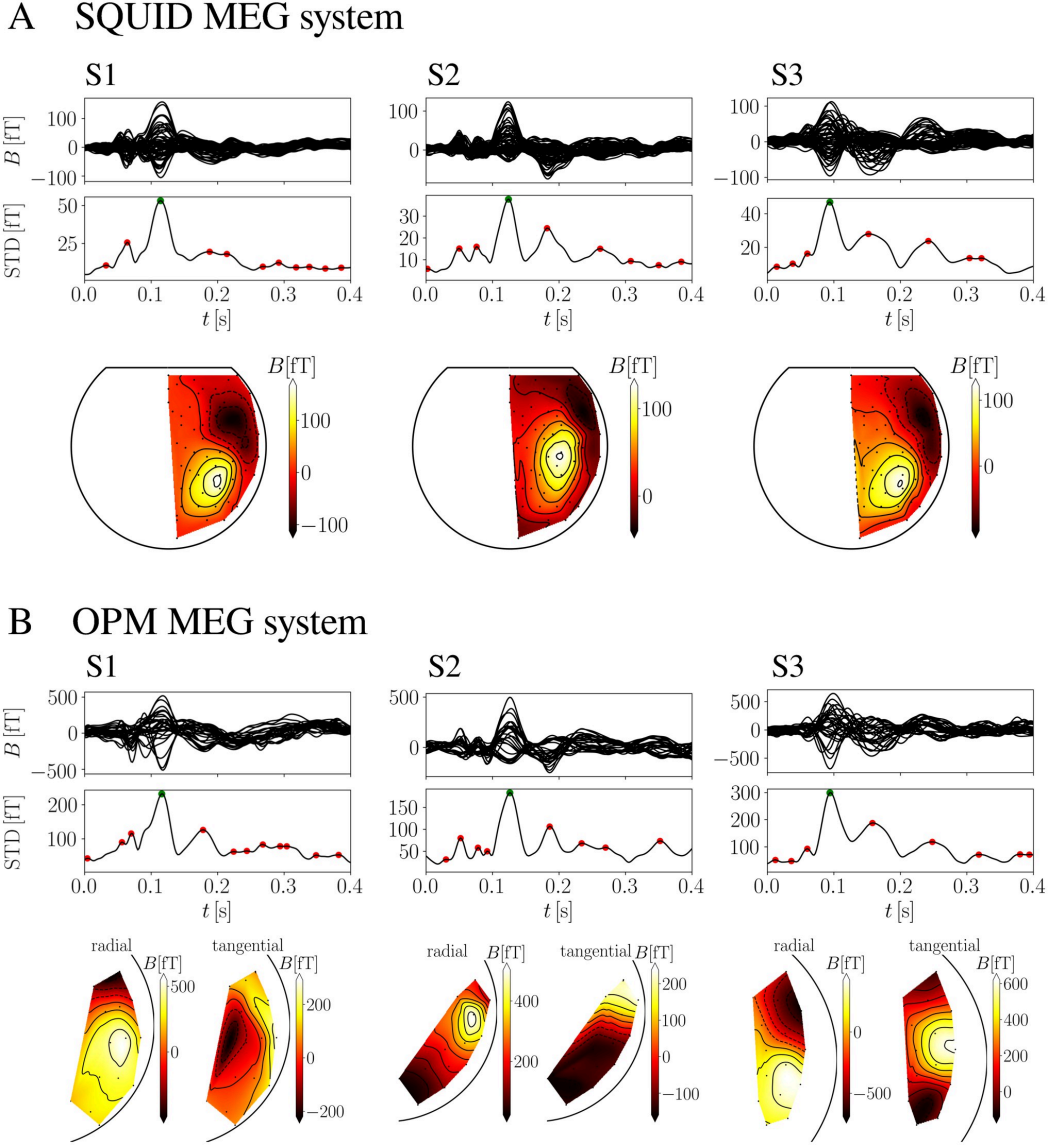
Display of measurements for three subjects using (A) SQUID and (B) OPM-MEG systems. The first row displays the butterfly plots of averaged magnetic flux density due to acoustic stimulation for individual measurements. The second row represents the STD(*t*), where the characteristic M100 peak is marked with a green dot. In the third row, the MFMs at the time of the M100 peak can be found. For the OPM-MEG system, we show MFMs for each measured component (radial and tangential) separately. For the SQUID system, we show only 62 channels covering the right hemisphere.

Fig 3 shows the results of preprocessed measurements of the auditory stimulation for both MEG systems (OPM and SQUID) for three subjects. Fig 3A shows the measurements obtained with the SQUID-MEG system. The upper three plots represent butterfly plots, i.e., averaged and filtered data on all measured channels. The middle three plots show the calculated STD function as defined in Eq (1). In this plot, we denote the identified local peaks with red dots, and the M100 peak with a green dot. On the bottom part, we show the contour plots, which indicate color-coded values of magnetic fields at all sensor locations. Brighter colors represent positive values of **B**, and darker colors negative values. The positions of the sensors on the contour plots do not represent real locations, but are projected on a 2D plane. Fig 3B shows the measurements obtained with the OPM-MEG system. The first two rows display butterfly plots and the STD function as in Fig 3A, while the bottom row shows contour plots for each component (tangential and radial). One can see that the responses on the butterfly plots and the times of M100 are very similar for both MEG systems. The dipolar pattern, which can be clearly detected for the SQUID system on the contour plots, can also be observed on the contour plots of the OPM-MEG system with only radial components.

**Fig 4:**
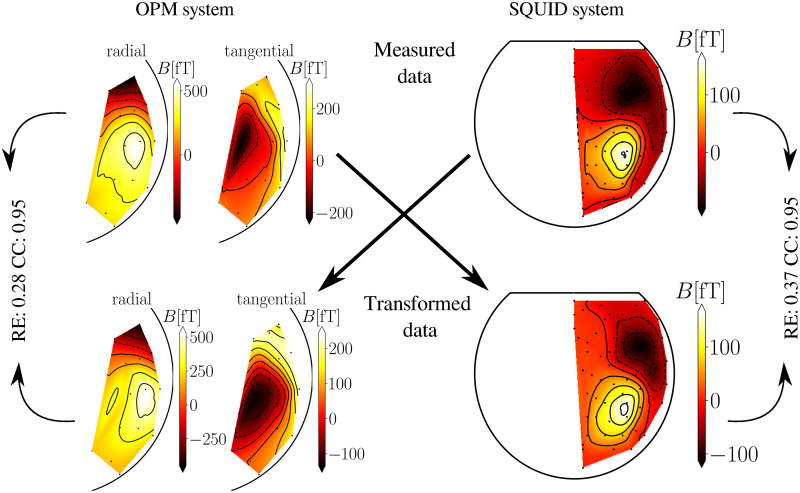
Transformation of the measured data between SQUID- and OPM-MEG for subject 2. The upper part shows the originally measured OPM and SQUID MFMs at the time of the M100 peak for one subject. The bottom part shows MFMs transformed to the other MEG layout. For the transformation, we used the MNE-BEM method. Arrows indicate which measured MFM was used in the transformation. RE and CC were calculated between individual pairs of the original and the transformed data. For the SQUID data, we used only the right half of the channels.

**Fig 5:**
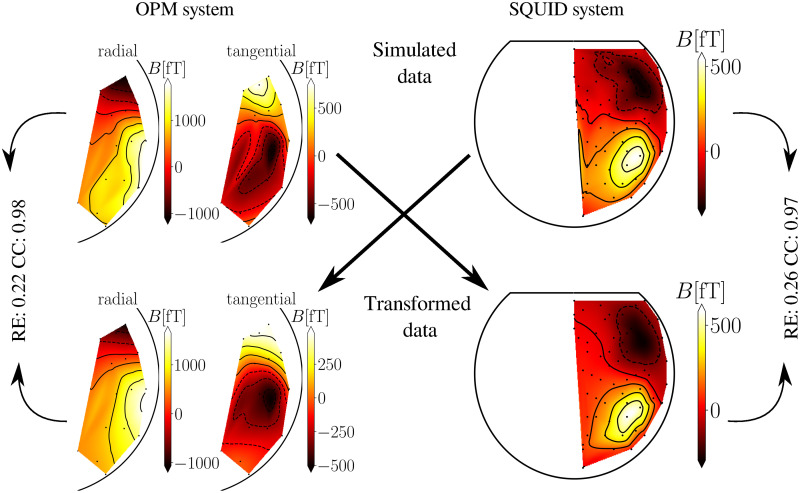
Transformation of the simulated data between SQUID- and OPM-MEG. The upper part shows the originally simulated OPM and SQUID system data. The bottom part shows the transformed MFMs. For the simulations, the noise level was determined by calculating the average SNR of the measured data for each system (SNR_OPM_ = 11.1 dB, SNR_SQUID_ = 13.8 db). Further details are identical to Fig 4.

Next, we show MFMs of the measured, simulated, and transformed data between the two MEG systems with the transformation method MNE-BEM in Figs 4 and 5 for one subject. All maps of measurements are shown for the time of the identified M100 peak. On the upper part (Fig 4), we show an example of the measured OPM and SQUID data. On the bottom part (Fig 4), we show an example of the transformed data from the SQUID and OPM to the OPM and SQUID-MEG system. We compared these MFMs by calculating RE (Eq (5)) and CC (Eq (6)), which are indicated next to the figures. We also show a transformation example for the simulated data (Fig 5). On both images one can see that the peaks of the maxima and minima coincide between the original and the transformed MFMs, so the CC value is noticeably high, implying good similarity between MFMs. The difference between the amplitudes between the transformed and the original data is noticeable and, consequently, the RE reflects this discrepancy. The noise level of simulations for all subjects was determined by averaging the values of SNR over all subjects for each MEG system separately. The average value of signal-to-noise ratio for all subjects was for the OPM-MEG system SNR_OPM_ = 11.1 dB, and for the SQUID-MEG system SNR_SQUID_ = 13.8 db. The values of SNR for each subject and each system are shown in Table 1. Additionally, we calculated the average value and the standard deviation.

**Table 1:** Calculated values of SNR for each subject and each MEG system.

	SNR[dB]			
Subject	OPM	OPM (rad.)	OPM (tan.)	SQUID
S1	13.7	13.8	13.6	15.8
S2	13.7	13.4	14.0	12.7
S3	10.0	10.1	10.0	13.9
S4	16.6	16.0	17.3	17.9
S5	10.7	13.1	9.1	14.2
S6	3.8	7.7	1.7	12.7
S7	5.4	7.2	4.2	14.8
S8	15.1	13.5	17.5	8.6
Average	11.1	11.9	10.9	13.8
Standard deviation	4.3	3.0	5.4	2.5

In this table we show the results of calculated SNR with Eq (2). Additionally, we calculated the values for each component (radial and tangential) separately.

**Fig 6:**
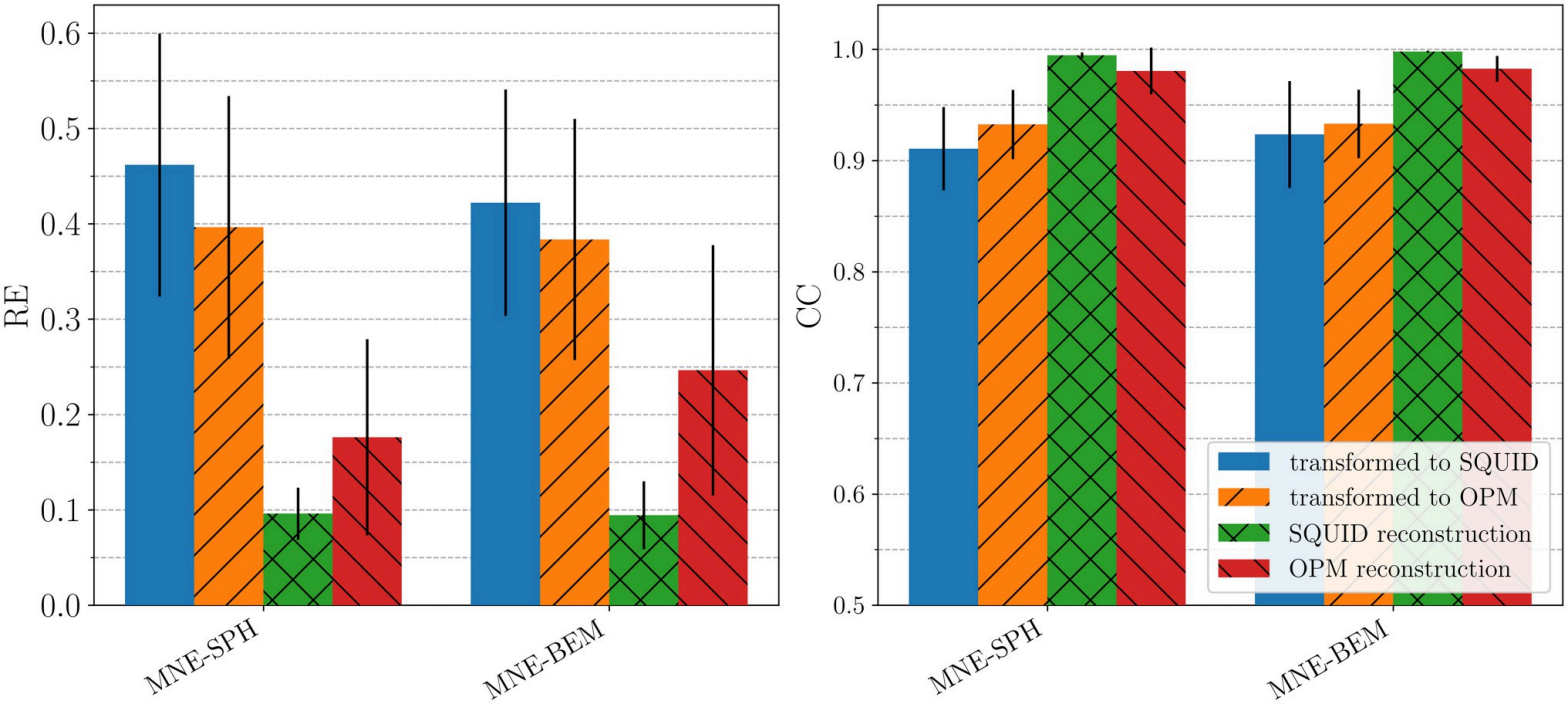
Comparison of two transformation methods for the measurements. The left image displays the relative error (RE), and the right the correlation coefficient (CC) between the measured original and the transformed magnetic field map from the other system. The orange column shows the results of the transformation of the SQUID data to the OPM-MEG system, and the blue column vice versa. The green and the red columns show the REs and CCs for comparison between the original and the reconstructed data. The black lines on top of the bars represent the standard deviation of the REs and CCs. Note that for both MNE methods the transformation cross-correlation CC is above 0.9, indicating that for the auditory M100 response both MEG devices record similar information.

Next, we tested different transformation techniques introduced in the Methods section using both the measured and the simulated data. First, we present the results of transformations of the measured data in Fig 6. The left subplot shows the averaged values of RE, and the right subplot the averaged values of CC for all subjects when comparing the MFMs at the time of the M100 peak. RE and CC were calculated for four cases: when comparing the measured SQUID data and the measured OPM data transformed to SQUID sensors (in the figure labeled as SQUID); when comparing the measured OPM data and the measured SQUID data transformed to OPM sensors (in the figure labeled as OPM); when comparing the measured and the reconstructed SQUID data, and when comparing the measured and the reconstructed OPM data. From Fig 6, one can see that the CC values of transformations are quite high for both methods, which indicates that the transformations were successful.

To further analyze the data presented in Fig 6, we did a statistical analysis for REs and CCs separately for all 8 subjects; the corresponding data set is in the (S2 Data). It was done with the statistical software GNU PSPP (https://www.gnu.org/software/pspp/). An independent t-test was run to determine if there were any differences in RE and CC between independent variable methods (MNE-SPH and MNE-BEM). No significant difference was found at 95% confidence interval for the parameter RE for the categories “transformed to SQUID” (blue bars in the left subplot: *t*(14) = −0.57, *p* = 0.576), and for “transformed to OPM” (orange bars: *t*(14) = −0.18, *p* = 0.861). No significant difference was found in the REs for “SQUID reconstruction” (green bars: *t*(14) = −0.10, *p* = 0.920), and for “OPM reconstruction” (red bars: *t*(14) = 1.11, *p* = 0.285). For the parameter CC, the results were the following: “transformed to SQUID” (blue bars in the right subplot: *t*(14) = 0.56, *p* = 0.582), “transformed to OPM” (orange bars: *t*(14) = 0.05, *p* = 0.965), “OPM reconstruction” (red bars: *t*(14) = −0.19, *p* = 0.850), and for the case “SQUID reconstruction” we found a significant difference (green bars: *t*(14) = 3.10, *p* = 0.008).

**Fig 7:**
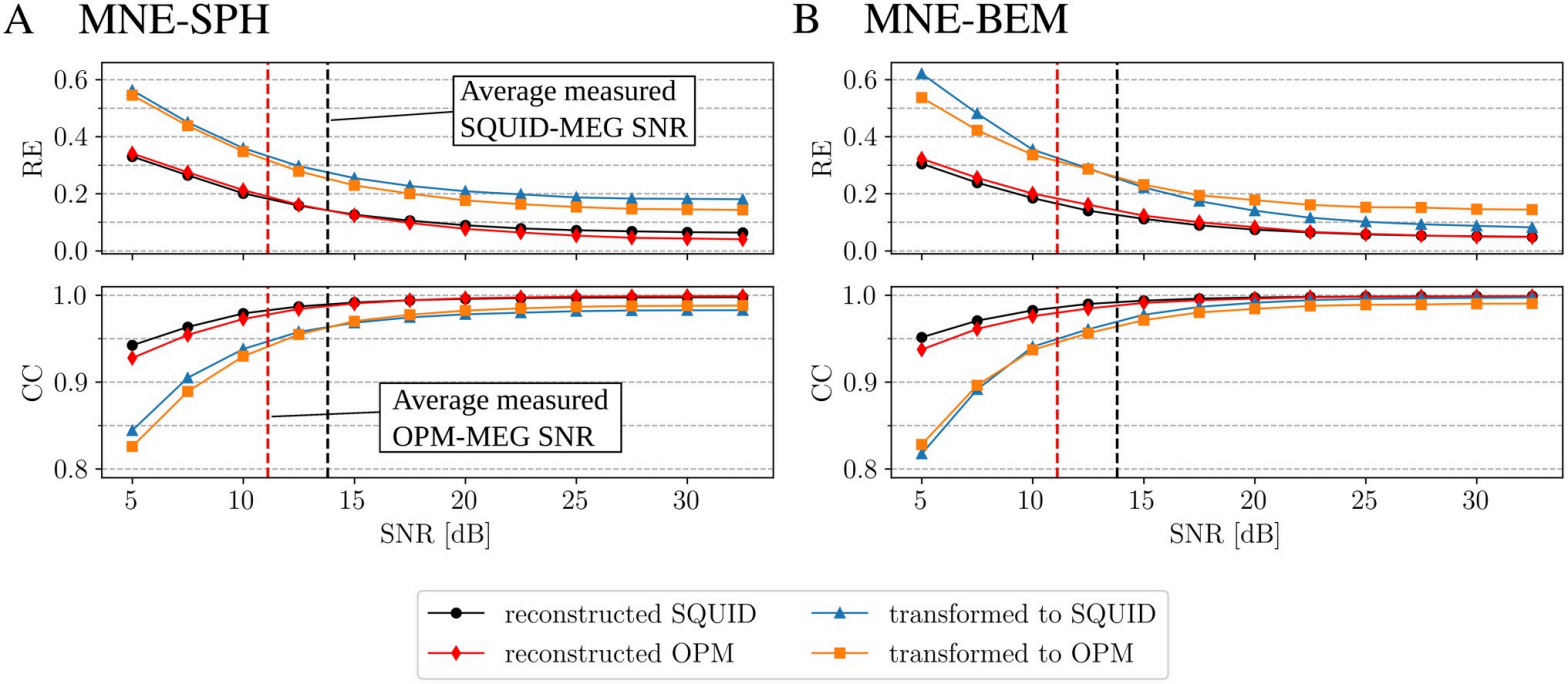
Transformation of simulated data using the method as indicated. Each subfigure shows the averaged RE and CC vs. SNR for four cases: comparison of simulated and reconstructed SQUID data; comparison of simulated and reconstructed OPM data; comparison of simulated SQUID data and transformed simulated OPM data to the SQUID system; comparison of simulated OPM data and transformed simulated SQUID data to the OPM system. Each subfigure is the result of averaging 10 times for different random added noise. The dashed vertical lines show average values of SNR, which were calculated from the measured data for each MEG system.

Then we present the results of different transformation methods of the simulated data in Fig 7: (A) MNE-SPH; (B) MNE-BEM. We checked the impact of channel noise levels (different SNR) on transformations. The figures of both subplots are the result of averaging 10 times for different added random noise. For high and low values of SNR (low and high noise levels), the methods performed similarly.

OPM sensors used in our OPM-MEG system measure two magnetic components, radial (normal to the head surface), and tangential (orthogonal to the radial component). We checked the impact of individual components of the OPM-MEG system on the error of transformation between SQUID and OPM systems for three configurations reflecting the experimental situation:

- Only the radial fields are known and, consequently, the transformation is between radial SQUID gradiometer data and radial OPM magnetometer data.
- Only the tangential OPM fields are known, and the transformation is between radial SQUID gradiometer and tangential OPM magnetometer data.
- Both OPM field directions are known, and the transformation is between radial SQUID gradiometer and combined radial and tangential OPM magnetometer data.

**Fig 8:**
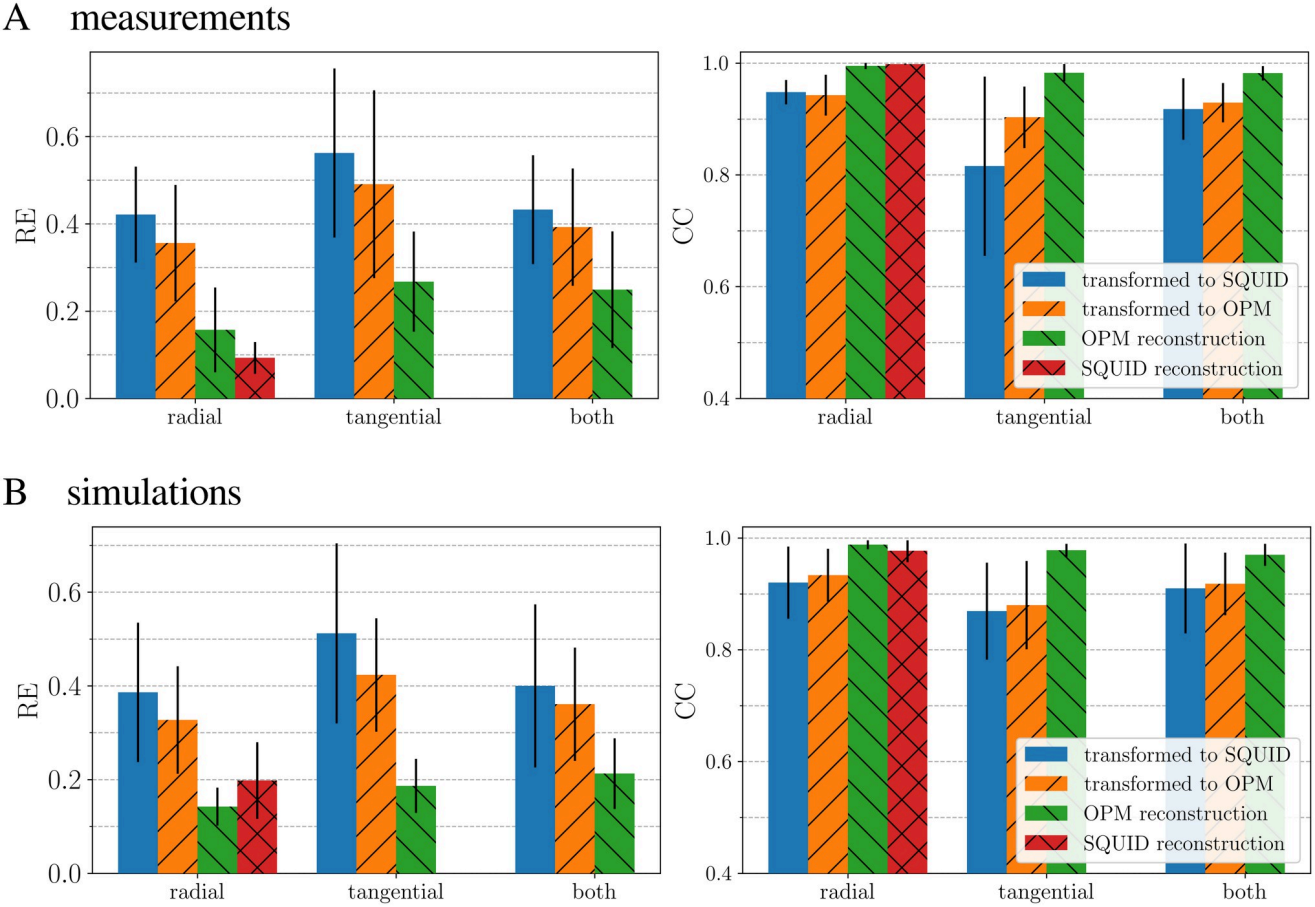
Impact of radial and tangential components during the transformation for the measured (A) and the simulated (B) MFM. The transformation was done using the MNE-BEM approach. We show three different transformation scenarios: using only radial or tangential, and both sensor components of the OPM-MEG system. The blue bars show the averaged RE and CC when transforming the OPM measurements to the SQUID sensors, and the orange bars vice versa. The green and the red columns show REs and CCs for reconstructed data. The black lines on the top of the bars represent the standard deviation of REs and CCs. The subfigures for simulations were averaged 10 times for different random added noise. The noise level for each system was SNR_OPM_ = 11.1 dB, and SNR_SQUID_ = 13.8 db. In both cases, (A) and (B), the best transformation results are obtained when using only the radial component.

The results for the measured and the simulated data are displayed in Fig 8. For the transformation, we used the MNE-BEM method. The subfigure of simulation is the result of averaging 10 times for different added random noise. The results show that the error is the greatest (large RE and small CC) when only the tangential component of OPM sensors is used.

To further analyze the results of measurements and simulations presented in Fig 8, we performed a one-way ANOVA for REs and CCs separately, for all 8 subjects. Using the GNU PSPP software, a statistical analysis was performed for the independent variable array configuration (radial, tangential and both OPM components). The corresponding data set is in the (S2 Data). For measurements (Fig 8A) we calculated the results for two categories “transformed to SQUID” and “transformed to OPM” separately. No significant difference was found between variables for REs of “transformed to SQUID” (blue bars on the left subplot, *p* = 0.167) and “transformed to OPM” (orange bars on the left subplot, *p* = 0.250) and CCs “transformed to OPM” (orange bars on the right subplot, *p* = 0.171). On the other hand, there was a significant difference for CCs of “transformed to SQUID” (blue bars on the right subplot, *p* = 0.041). Therefore, a post-hoc analysis was run to determine the difference between the groups. For the Fisher’s least significant difference (LSD) test, values are below or near *p* = 0.05 for the following comparisons: using only tangential and only radial components (*p* = 0.018); using both and only radial components (*p* = 0.052). For the Bonferroni test, which is corrected for multiple comparisons, only one value is near *p* = 0.05: comparing the cases using only tangential and only radial components (*p* = 0.053). Next, we show the results for the category “reconstructed OPM”. REs do not differ significantly (green bars on the left subplot, *p* = 0.195). On the other hand, for CCs this value is (green bars on the right subplot, *p* = 0.076), therefore we ran a post-hoc analysis. When using the Fisher’s LSD, we found a significant difference between “both OPM directions” and “radial directions” (*p* = 0.044), and between “radial directions” and “tangential directions” (*p* = 0.054). When using the more conservative Bonferroni test, no significant difference was found.

For the simulations (Fig 8B), we first present the results for the categories “transformed to SQUID” and “transformed to OPM” (color bars same as in the previous paragraph). No significant difference was found between variables for REs of “transformed to SQUID” (*p* = 0.345), “transformed to OPM” (*p* = 0.329), and CCs “transformed to SQUID” (*p* = 0.446), “transformed to OPM” (*p* = 0.277). When we performed an analysis for the category “reconstructed OPM”, the significance value was for REs *p* = 0.104 and CCs *p* = 0.072. The post-hoc analysis for REs when using the Fisher’s LSD test showed a significant difference only between “both OPM directions” and “radial directions” (*p* = 0.037). When using the Bonferroni test, no significant difference was found. The post-hoc analysis for CCs when using the Fisher’s LSD test showed a significant difference only between “both OPM directions” and “radial directions” (*p* = 0.023). For the Bonferroni test, this value was (*p* = 0.070).

## Discussion

The use of OPM magnetometers in MEG is gaining in popularity. Although some works already show whole-head coverage OPM systems [48, 49], most laboratories still operate with a limited number of sensors, and therefore planning suitable layouts adapted to the physiological question of interest is very important. If prior information of the brain activity is known before the measurements, a layout can be calculated using a selection algorithm [50]. In our work, we present two approaches to obtain exemplary MFMs for AEFs as the basis for OPM sensor layouts: 1. Transform existing data from a SQUID system to a hypothetical layout of OPM sensors; 2. Generate a forward model and place sources in regions of interest and simulate the resulting MFM for a specific sensor layout. The first approach can be used to qualitatively compare the measurements obtained by two different MEG systems (in our case, OPM-MEG and standard SQUID-MEG systems). The algorithm to transform data from one system to another works on the principle of calculating source reconstruction, and then calculating the magnetic field on the other system using a forward solution. In this study, we evaluate different transformation methods on measured and simulated auditory response MFMs. In the transformations, we used only the right hemisphere channels of the SQUID-MEG channels, as the OPM sensors were placed over the right hemisphere to measure the field due to currents in the right auditory cortex. For each subject and both MEG systems, we calculated the values of SNR averaged for all system channels (Table 1). The results show that, on average, the SQUID-MEG system has higher SNR than the OPM-MEG system. This result contradicts the theoretical works [22, 23], which show that the OPMs should have a much better SNR compared to the SQUIDs due to the reduced distance between sensors and the brain. We hypothesize that the main reason for this discrepancy is the fact that the sensors are moving in relatively high gradient fields [48]; contrary to the OPMs, SQUIDs are static. Every minor subject’s head movement results in relatively high measured fields; this should be further explored in future work. The measurement setup in this work uses triple-axis compensation on the outside surfaces of the Ak3b, which ensures that the sensors operate inside the linear regime [35, 36]. This is true for very small movements (≈ 4mm). Using a more sophisticated active shielding such as in [51, 52] would increase SNR and allow for bigger movements [10, 53]. Another reason for these SNR results is the fact that the OPMs are configured as magnetometers and the SQUIDs as gradiometers, whose role is to reduce external magnetic noise [2, 54]. Additionally, we calculated SNR for each component of the OPM-MEG system separately. We obtained higher values of SNR for the radial components.

In our work, we used two implementations of the MNE source reconstruction. One uses the spherical volume conductor forward model (MNE-SPH), and the other an individual geometry of each subject (MNE-BEM). Both transformation methods give slightly different results when comparing the measured and the transformed MFM (Fig 6). Overall, MNE-BEM performs slightly better than MNE-SPH; this has already been predicted in one of the previous works [19]. To further explore how the results between the two transformation methods differ, we performed an independent samples t-test. The only significant difference was found when comparing the CCs of the SQUID system reconstruction (green bars in the right subplot of Fig 6).

The results for the simulated data in Fig 7 support the results from the experimental data. We checked the effect of noise added to the simulation for each transformation method. Both methods, MNE-SPH and MNE-BEM, perform similarly very well. For low values of SNR, MNE-SPH performs a little better, and for high values of SNR, MNE-BEM has a lower transformation error. The simplified implementation of the MNE-SPH method has one advantage over the MNE-BEM method, it does not require the real geometry of each individual (BEM). From this we can conclude that for the transformation of MFMs between different MEG systems, the use of the MNE-SPH method with the simplified volume conductor model and the simplified source space is sufficient.

For a special case of focal sources, like AEF, an equivalent current (ECD) model can also be used for source localization. We have checked if such a simplified source model can be used for the transformation of data between different MEG systems. Details can be found in S3 Appendix. Results show that for focal sources, a simplified ECD model can also be used for data transformation; however, the MNE source model is preferred in general use.

Since current OPMs allow measuring two orthogonal directions of the magnetic field, it is important to study how each field component impacts the quantitative transformation result. Generally, this topic is not new as the SQUID-MEG systems of the TRIUX^™^ neo type (https://megin.fi/triux-neo/) measure a tangential gradient of the radial field [55], and the Yokogawa and CTF systems measure radial gradients. A triple-axis SQUID device was discussed in [56]; in their work they showed that quasi-radial dipolar activity can be reconstructed from data measured with a MEG system with three measuring components. Recently, the advantages of a triple-axis OPM-MEG system have also been studied [24]. Since MNE-BEM is the most refined method in this study, the directionality analysis was performed for the experimental and the simulated data (Fig 8). The best transformation results for the measured data (lowest RE and highest CC) are obtained when only the radial component is used. We obtain the worst transformation (highest RE and lowest CC) results when only the tangential component is used. The transformation error of using both components is slightly higher than for the radial component only. To further analyze how the results between the groups (using only radial, only tangential, and both OPM-MEG components combined) differ, we performed a one-way ANOVA. The results show that for some cases of RE and CC, there is a significant difference between groups. We conjecture that the best result is obtained for the radial-component-only configuration due to the SQUID system used here, which measures the radial gradient. We hypothesize that the tangential component contains field information that is “hidden” to the radial component.

The transformation results for the simulated data in Fig 8B are very similar to the measurement results in Fig 8A, and any differences are not significant if the error bars are considered. These results are in accordance with a simulation study where it is argued that the radial component is a better choice than the tangential component for measurements in MEG [23]. One reason why transformation from SQUID data to OPM data works better than vice versa is probably the low number of OPM sensors. In this study, we simultaneously measured with 15 sensors (30 channels). An increased number of OPM sensors would give us more information on the measured phenomenon, and transformation quality is likely to be more symmetric [57].

The successful transformation between SQUID-MEG and OPM-MEG of the AEF M100 demonstrates that a 15-sensor dual-axis OPM magnetometer system records similar information content as a 62-channel SQUID gradiometer for this simple application. Clearly, the 62 SQUID gradiometers cover a much larger area than the 15 OPM magnetometers, but a simple choice of placement apparently makes it possible to extract similar information. Obviously, prior information was used to place the OPM sensors; nevertheless, prior information did not use individual anatomy, but rather general knowledge that the AEF M100 shows large amplitudes over central to temporal brain regions.

For future work it would be worth testing these transformation techniques between measurements made with full head coverage OPM- and SQUID-MEG systems. Either the transformation errors would decrease, or alternatively, the transformation could fail if a full head coverage OPM-MEG detected information not visible in SQUID-MEG. Then the transformation methods would be a test tool to prove additional information in OPM-MEG. It would also be interesting to test the transformation with a SQUID system, which has multiple measuring components.

The transformation method presented in this work could be used to plan measurements with OPM-MEG systems if prior SQUID-MEG results are available. The performance of source reconstruction and spatial resolution of an OPM-MEG system with a limited number of sensors is linked to the layout of sensor placement around the head [58]. An advanced method for selecting an optimal layout could be the statistically based lead selection algorithm, which was developed for the purpose of electrocardiography [50], and magnetocardiography [57]. We argue that this method could be applied to OPM-MEG when a large database of the magnetic fields on all possible placements around the head is available. The transformation method presented in this study could be used to harvest a large pool of SQUID measurements made in the past.

## Conclusion

This study addresses the comparison and transformation of results from SQUID and OPM-based MEG systems. We presented a framework for the transformation of measurements between the MEG systems using inverse and forward models serving as an intermediate representation of the measured data.

We applied two transformation methods, MNE-SPH and MNE-BEM. In general, both methods yield very good results when comparing measurements and simulations. We also studied the influence of radial and tangential field components on the transformation error. We have shown that the transformation of tangential OPM channels to radial SQUID channels yields a larger error compared to the transformation between radial OPM to radial SQUID channels. Interestingly, combining tangential and radial OPM channels does not lower the transformation error.

In this work, we demonstrated that AEF measurements on one hemisphere with 15 OPM dual-axis sensors generate comparable data to the measurements with SQUID sensors covering a similar hemispheric area. The successful AEF M100 transformation shows that an OPM-MEG at costs considerably lower than a SQUID-MEG can yield similar information if full head coverage is not required.

## Supporting information

S1 Appendix Experiment of the dual-axis sensor orthogonality. In this appendix we explain in detail an experiment where we measured the OPM’s dual-axis sensor orthogonality.
(PDF)

S2 Appendix Detailed description of the transformation methods. We present two forward models (magnetic fields outside a spherically symmetric conductor and outside a multi-layer shell conductor). The MNE-SPH and MNE-BEM methods are also explained in detail.
(PDF)

S3 Appendix Transforming MEG data using an equivalent current dipole (ECD) fit approach. Magnetic field maps which have a dipolar-like pattern can also be transformed by solving the source reconstruction with an ECD fit. In this appendix, we show the methods and results of transforming AEF measurements and simulations.
(PDF)

S1 Data Files containing the averaged measured and simulated data. Individual files are in.fif format and can be read using the MNE-Python software package.
(ZIP)

S2 Data Files containing the results (REs and CCs) of individual subjects for Figs 6 and 8. Individual files are spreadsheets in .ods format and can be read using a standard word processor.
(ZIP)

S1 Table Acronyms and abbreviations. This table lists all the acronyms and abbreviations that appear in the text at least three times with full names.
(PDF)
