# Supplementary material for: Transforming and comparing data between standard SQUID and OPM-MEG systems
Source: PLoS One. 2022 Jan 19;17(1):e0262669. doi: 10.1371/journal.pone.0262669 (PMC8769297; doi:10.1371/journal.pone.0262669)
Supplement: S1 Appendix — In this appendix we explain in detail an experiment where we measured the OPM’s dual-axis sensor orthogonality. (PDF) [file pone.0262669.s001.pdf]

S1 Appendix. Experiment of the dual-axis sensor orthogonality.

In our study, the intrinsic orthogonality between the sensor axes is of importance and it was determined experimentally. Five optically pumped magnetometers (OPMs) of type QZFM Gen-2 (made by the company QuSpin [1]) were oriented at right angles towards each other in a holder as shown in Fig 1 with two sensors pairs measuring each the same direction of field. This holder was placed in a triple axis Helmholtz-like coil system made of printed circuit board coils with a field homogeneity better than 1 % in the central region. The coil and the sensor holder are shown in Fig 2. The sensor holder is placed on a cross shaped base and this base can be aligned with a ruler against the coil axes and an angle mismatch of less than 2 degrees was achieved. The coil was driven at 11.5 Hz with a current generating the magnetic field of approximately 1 nT in the central region. The OPMs were operated in the dual-axis mode and the six signals were recorded simultaneously. A 4 s section of data is shown in Fig 3, where the signals from the OPM axes orthogonal to the field were multiplied by a factor of 40 as indicated in Fig 3. Note as well the different scale of the signals as indicated by the red range arrow. Since the amplitude of the signal in the field direction and the amplitude of the multiplied signal in the orthogonal direction are similar it can be concluded that the orthogonality is better than 2.5 % for all three sensors. The slow background variation of the orthogonal signals is the background variation of the field in our magnetically shielded room with active compensation running. It is not identical for all three sensors as the sensors are oriented at right angles and detect a slightly different background field. The procedure was repeated for several frequencies and several field magnitudes and similar results were obtained.

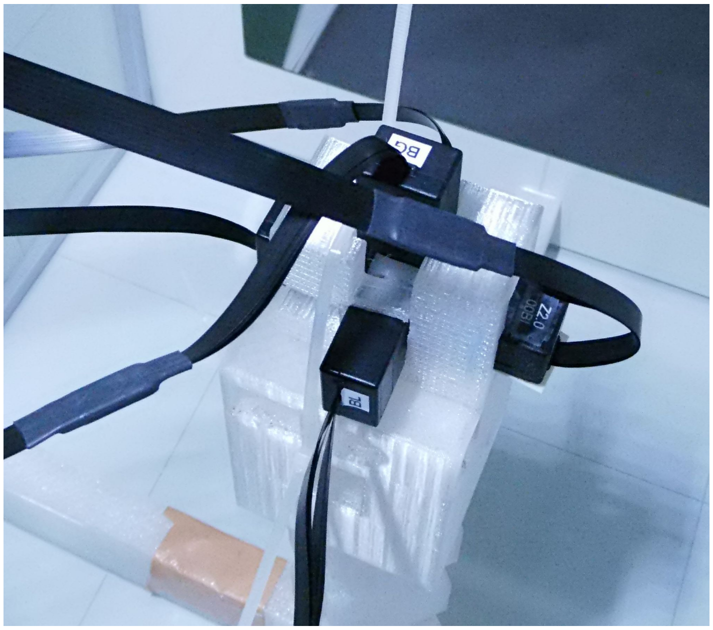

Fig 1. Picture of the 3D printed sensor holder for testing the sensor orthogonality. The OPM sensors, which we tested, are placed in the sensor holder.

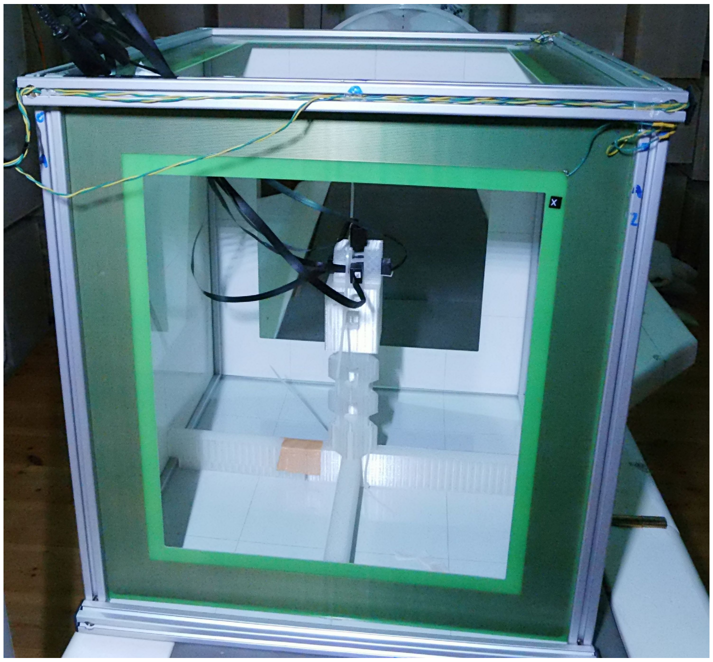

Fig 2. Picture of the Helmholtz-like coil system. The sensor holder with OPM sensors is situated inside the coil system.

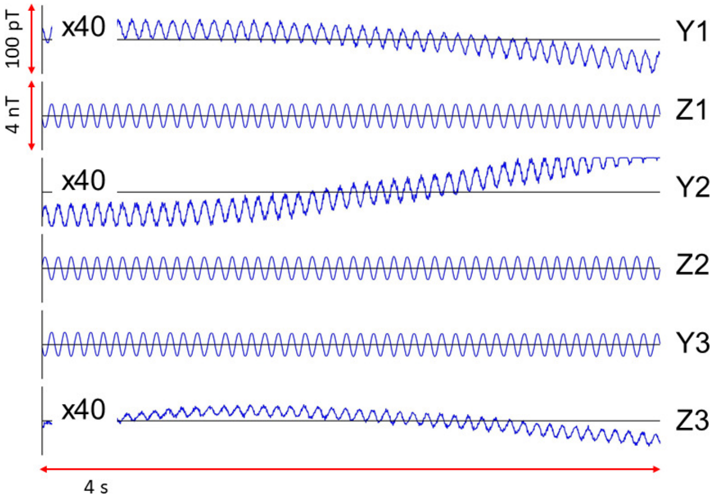

Fig 3. Measured signals during the orthogonality experiment. 4 s data set is presented. The signals Z1 and Z2 measure in the direction of the coil field, whereas the orthogonal signals Y1 and Y2 are 40 times weaker.

References

1. Osborne J, Orton J, Alem O, Shah V. Fully integrated, standalone zero field optically pumped magnetometer for biomagnetism. In: Shahriar SM, Scheuer J, editors. Steep Dispersion Engineering and Opto-Atomic Precision Metrology XI. San Francisco, United States: SPIE; 2018. p. 51.
