# Supplementary material for: Transforming and comparing data between standard SQUID and OPM-MEG systems
Source: PLoS One. 2022 Jan 19;17(1):e0262669. doi: 10.1371/journal.pone.0262669 (PMC8769297; doi:10.1371/journal.pone.0262669)
Supplement: S2 Appendix — We present two forward models (magnetic fields outside a spherically symmetric conductor and outside a multi-layer shell conductor). The MNE-SPH and MNE-BEM methods are also explained in detail. (PDF) [file pone.0262669.s002.pdf]

S2 Appendix. Detailed description of the transformation methods.

Magnetic fields outside a spherically symmetric conductor.

The analytical solution for the magnetic field ($\vec{B}$) outside the volume conductor was derived by Sarvas [1]:

$$\vec{B}(\vec{r}) = \frac{\mu_0}{4\pi F^2}\Big(F(\vec{p}\times\vec{r}_p) - (\vec{p}\times\vec{r}_p\cdot\vec{r})\nabla F\Big), \tag{1}$$

$$F = |\vec{a}|\Big(|\vec{r}||\vec{a}| + \vec{a}\cdot\vec{r}\Big), \tag{2}$$

$$\nabla F = \left(\frac{F}{|\vec{a}|^2} + |\vec{a}| + |\vec{r}|\right)\vec{a} + \left(\frac{|\vec{a}|^2}{|\vec{r}|} + |\vec{a}|\right)\vec{r}, \tag{3}$$

where $\vec{a} = \vec{r} - \vec{r}_p$, $\vec{r}_p$ denotes the location and $\vec{p}$ the direction and strength of the equivalent current dipole (ECD) inside the spherical volume conductor, $\vec{r}$ represents the location of the sensor. A schematic representation of these quantities is shown in Fig 1. Note, that in this model the radial component of the ECD yields no magnetic field outside the sphere. The calculated magnetic field does not depend on the conductivity and size of the sphere, only the position of the sphere matters.

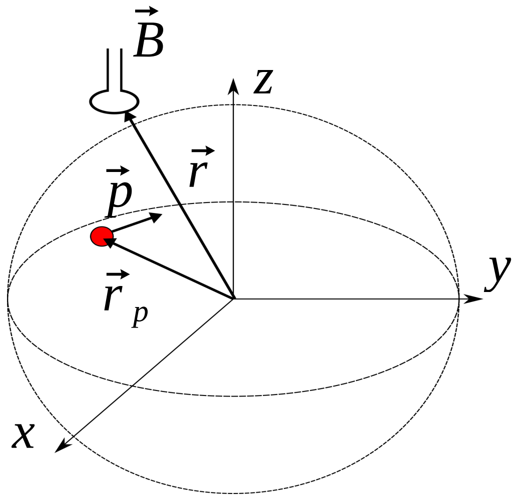

Fig 1. Schematic representation of a source inside a spherical volume conductor. In the figure we schematically presented all the quantities that are required to calculate the magnetic field outside a conductive sphere using the Eq. 1.

Magnetic fields outside a multi-layer shell conductor.

The forward problem in the software package MNE-Python can be solved by using a more complex conductor model. The model that we used is a source inside a conductor with piecewise homogeneous subregions G_i ($i = 1, \ldots, m$). Each G_i has a constant conductivity $\sigma = \sigma_i$. We used a 3 layer model ($m = 3$) in our calculations. The total

current density is divided into the primary and the volume current density ($J = J_p + J_v$). The magnetic field for this case can be calculated using the Geselowitz formula [2]:

$$\vec{B}(\vec{r}) = \vec{B}_0(\vec{r}) - \frac{\mu_0}{4\pi}\sum_{i=1}^{m}(\sigma_i^- - \sigma_i^+)\int_{S_i}\phi(\vec{r'})\frac{\vec{r}-\vec{r'}}{|\vec{r}-\vec{r'}|^3}\times \mathrm{d}\vec{S'}, \tag{4}$$

where

$$\vec{B}_0(\vec{r}) = \frac{\mu_0}{4\pi}\int_V \vec{J}_p(\vec{r'})\frac{(\vec{r}-\vec{r'})}{|\vec{r}-\vec{r'}|^3}\times \mathrm{d}\vec{V'} \tag{5}$$

is the magnetic field of $\vec{J_p}$ in an infinite homogeneous medium, S_i is the surface of the i-th subregion and ϕ is the electric potential on that surface. This formula cannot be calculated analytically, we have to first solve the electrical problem to obtain the potential ϕ on border surfaces between subregions of different conductivities. A preferred method is to use the boundary element method. Contrary to the simplified model of sources inside the spherical volume conductor Eq. 1, radial components of the sources produce magnetic fields outside the volume conductor and also the conductivities of the individual layers affect the calculated magnetic field.

Detailed description of the minimum norm estimate (MNE) method using a homogeneous spherical volume conductor model and source space distributed on a sphere (MNE-SPH).

This method uses an implementation of the MNE method as described in [1], working on similar principles as the MNE method implemented in MNE-Python (explained below), but with some differences. MNE method is generally based on the selection of a source space, consisting of a large number of current dipoles, and the forward solution, which calculates the magnetic field at the sensor locations from the dipoles in the selected source space. The forward solution in MNE-SPH uses a simplified model of the current dipole source inside a homogeneous spherical volume conductor (Eq. 1). The sensor model in this implementation did not take into account the real sensor geometry. We calculated the magnetic field in the center of the gas of cell of the optically pumped magnetometer (OPM) and each pick up coil of the superconducting quantum interference device (SQUID) gradiometer.

The source space consists of dipoles located on the upper part of a sphere that best fits the outer brain surface. The exact locations are the chosen centers of faces of an upsampled icosahedron with 320 faces and 162 vertices. In our case, we used the top 275 faces. To construct this upsampled icosahedron, we recursively subdivide the regular icosahedron, in our case we did two iterations. The radius and center of the icosahedron were chosen individually for each subject depending on the coregistration between the head and the magnetoencephalography (MEG) system. To solve the minimum norm estimates, we used the singular value decomposition method (SVD) [3]. Since the inverse problem is ill-posed, we have to regularize it [4].

Next, we explain all the major steps of our MNE implementation. First, we calculated the lead fields $\vec{L}$, we obtain them by solving the forward problem (Eq. 1). At the i-th sensor location with sensor orientation $\vec{q_i}$ we measure the magnetic field (B_i):

$$B_i = \vec{B}(\vec{r_i}) \cdot \vec{q_i}. \tag{6}$$

Using 1 and 6 we can calculate B_i for m sources in the source space as:

$$B_i = \sum_{j=1}^{m}\vec{L}_{i,j}\cdot\vec{p}_j, \tag{7}$$

where

$$\vec{L}_{i,j} = \frac{\mu_0}{4\pi F^2}(F(\vec{r}_{pj} \times \vec{q}_i) - \nabla F \cdot \vec{q}_i(\vec{r}_{pj} \times \vec{r}_i)). \quad (8)$$

The lead field matrix $\mathbf{\Gamma}$, which is a linear operator that calculates the magnetic field from the known source distribution is then defined as:

$$\mathbf{\Gamma}_{kl} = \mathbf{L}_k \cdot \mathbf{L}_l, \quad (9)$$

where $\mathbf{L}_i = (\vec{L}_{i,1}, \dots, \vec{L}_{i,n})$ and n is the total number of sensors. To calculate its inverse, we used the SVD method $\mathbf{\Gamma} = \mathbf{U\Lambda V}^{\mathrm{T}}$ implemented in the Python software package NumPy [5]. During this step, we used a simplified truncated SVD approach for regularization of the diagonal matrix $\mathbf{\Lambda}$ to obtain $\tilde{\mathbf{\Lambda}}$. First, we searched for the largest value $\max(\mathbf{\Lambda})$ then we made every element smaller than λ_r equal to $\Lambda_{i,i} = 0$, where $\lambda_r = (1/40)\max(\mathbf{\Lambda})$. The Moore-Penrose inverse of matrix $\mathbf{\Gamma}^{-1}$ is then defined as $\mathbf{\Gamma}^{-1} = \mathbf{V}\tilde{\mathbf{\Lambda}}^{-1}\mathbf{U}^{\mathrm{T}}$ [6]. To calculate the weight $\mathbf{w}$, we used the relation $\mathbf{w} = \mathbf{\Gamma}^{-1}\mathbf{B}$. With the weight $\mathbf{w}$ and all the lead fields $\vec{L}$, we can calculate the strength of all three components for the j-th dipole in the source space:

$$\vec{p}_j = \sum_{i=1}^{n} \mathbf{w}_i \vec{L}_{i,j}, \quad (10)$$

Using the solution $\vec{p}_j$, we can calculate the magnetic field on another MEG system, but we need to calculate the lead fields $\vec{L}'$ of the MEG system to which we want to transform. The magnetic field $\mathbf{B'} = (B'_1, B'_2, \dots, B'_n)$ is then calculated using the equation 7.

Detailed description of the MNE method using subject's individual boundary element method (BEM) model (MNE-BEM).

This method uses the core functions implemented in the software package MNE-Python, all details are accessible in [7] and [8]. The source reconstruction in this method uses individual geometry for each subject (BEM model). We calculated the source space, which is surface-based and is distributed over the outer surface of both hemispheres. Each hemisphere has around 4000 vertices. Each vertex represents a current dipole with a fixed orientation, which is perpendicular to the brain's surface. The forward solution in MNE-BEM uses an individual 3-layer BEM model (outer scalp layer, inner scalp layer, outer brain layer). For BEM calculations the linear collocation method [9] with isolated skull approach [10] is used. The sensor model in this implementation takes into account the real sensor geometry. The output of the individual MEG channels is approximated by integrating over a set of points inside the sensor. For the OPM sensors, the magnetic fields are calculated for 8 points with the same weights. The exact geometry for the QuSpin SERF OPM is provided by the MNE-Python in the OPM example dataset. For the SQUIDs, the magnetic field is calculated for 4 points of each (two) pick up coil of the gradiometer. The sensor geometry for this SQUID system is built in the software MNE Python. In MNE-Python, the inverse operator is calculated with the forward model and the noise covariance matrix. The noise covariance matrix represents the measure of the noise level in channels and it is used in the inverse solution to define which lead fields add more weight to the solution (less noisy channels have greater power), as well as in the regularization of the solution. According to the package documentation of MNE-Python, the larger values of regularization parameter λ correspond to spatially smoother and weaker current amplitudes, whereas smaller values lead to the opposite [8]. We used the default regularization parameter in MNE-Python ($\lambda = 1/9$). All detailed information on MNE can be found in the documentation of the

software package MNE Python on its web page https://mne.tools/stable/overview/index.html.

The exact commands that we used in our code to transform the data from one system to another and vice versa are shown on Fig 2. First, we import the averaged data, which we previously preprocessed, we crop it to have only the data of the M100 peak, which we want to transform. Next, we compute the forward solution and then import the noise covariance matrix which we calculated during the preprocessing. Then we calculate the inverse operator. In our work we used fixed dipoles (direction of each dipole is oriented normal to the brain surface), therefore we set the parameters as depth=None, loose=0, fixed=True. These parameters can be changed to have more "free" orientations, we found our configuration the best to work with, more details can be seen on the package documentation website. Then we apply the inverse operator to the data to obtain the source estimate, i.e., the parameters of each current dipole ($\vec{P}_i$) in the source space. Like in the MNE-SPH method, the transformation of data to the other MEG system is done by applying (7) using lead fields ($\vec{L}'$) of the MEG system, to which we want to transform.

```
import mne

# import the averaged Evoked data object
squid_evoked = mne.Evoked(evoked_squid_path)
opm_evoked = mne.Evoked(evoked_opm_path)

# get only the data of M100 (peak_opm is the M100 time for the OPM MEG system)
opm_evoked = opm_evoked.crop(peak_opm, peak_opm)
squid_evoked = squid_evoked.crop(peak_squid, peak_squid)

# compute the forward operator, for the opms we need a sensor definition file (coil_def_
# fname), fname_trans is the transformation file from head to MRI, src is the source space,
# bem_sol is the BEM solution
with mne.use_coil_def(coil_def_fname):
    fwd_opm = mne.make_forward_solution(opm_evoked.info, fname_trans_opm, src, bem_sol,
                                        ignore_ref=True)
fwd_squid = mne.make_forward_solution(squid_evoked.info, fname_trans_squid, src, bem_sol,
                                      ignore_ref=True)

# read the noise covariance matrix, which was calculated during the preprocessing step
noise_cov_opm = mne.read_cov(noise_covariance_opm_path)
noise_cov_squid = mne.read_cov(noise_covariance_squid_path)

# compute the inverse operator, the parameters depth, loose and fixed can be changed
inv_squid = mne.minimum_norm.make_inverse_operator(squid_evoked.info, fwd_squid,
                                   noise_cov_squid, depth=None, loose=0, fixed=True)
inv_opm = mne.minimum_norm.make_inverse_operator(opm_evoked.info, fwd_opm,
                                   noise_cov_opm, depth=None, loose=0, fixed=True)

# apply the inverse operator and calculate the the source estimates
stc_squid = mne.minimum_norm.apply_inverse(squid_evoked, inv_squid, lambda2=1/9,
                                           method="MNE", pick_ori=None)
stc_opm = mne.minimum_norm.apply_inverse(opm_evoked, inv_opm, lambda2=1/9,
                                         method="MNE", pick_ori=None)

# calculate the magnetic fields on the other system
transformed_opm_evoked = mne.apply_forward(fwd_opm, stc_squid, opm_evoked.info)
transformed_squid_evoked = mne.apply_forward(fwd_squid, stc_opm, squid_evoked.info)
```

Fig 2. Software code of the MNE-BEM method. Overview of the crucial MNE-Python functions used to transform measurements from one MEG system to the other.

References

1. Sarvas J. Basic mathematical and electromagnetic concepts of the biomagnetic inverse problem. Physics in Medicine and Biology. 1987;32(1):11–22. doi:10.1088/0031-9155/32/1/004.

2. Geselowitz D. On the Magnetic Field Generated Outside an Inhomogeneous Volume Conductor by Internal Current Sources. IEEE Transactions on Magnetics. 1970;6(2):346–347. doi:10.1109/TMAG.1970.1066765.

3. Golub GH, Reinsch C. Singular value decomposition and least squares solutions. Numerische Mathematik. 1970;14(5):403–420. doi:10.1007/BF02163027.

4. Willoughby RA. Solutions of Ill-Posed Problems (A. N. Tikhonov and V. Y. Arsenin). SIAM Review. 1979;21(2):266–267. doi:10.1137/1021044.

5. Harris CR, Millman KJ, van der Walt SJ, Gommers R, Virtanen P, Cournapeau D, et al. Array Programming with NumPy. Nature. 2020;585(7825):357–362. doi:10.1038/s41586-020-2649-2.

6. Ben-Israel A, Greville TNE. Generalized inverses: theory and applications. 2nd ed. No. 15 in CMS books in mathematics. New York: Springer; 2003.

7. Gramfort A, Luessi M, Larson E, Engemann DA, Strohmeier D, Brodbeck C, et al. MNE software for processing MEG and EEG data. NeuroImage. 2014;86:446–460. doi:10.1016/j.neuroimage.2013.10.027.

8. Gramfort A. MEG and EEG data analysis with MNE-Python. Frontiers in Neuroscience. 2013;7. doi:10.3389/fnins.2013.00267.

9. Mosher JC, Leahy RM, Lewis PS. EEG and MEG: forward solutions for inverse methods. IEEE Transactions on Biomedical Engineering. 1999;46(3):245–259. doi:10.1109/10.748978.

10. Hamalainen MS, Sarvas J. Realistic conductivity geometry model of the human head for interpretation of neuromagnetic data. IEEE Transactions on Biomedical Engineering. 1989;36(2):165–171. doi:10.1109/10.16463.
