# Supplementary material for: Transforming and comparing data between standard SQUID and OPM-MEG systems
Source: PLoS One. 2022 Jan 19;17(1):e0262669. doi: 10.1371/journal.pone.0262669 (PMC8769297; doi:10.1371/journal.pone.0262669)
Supplement: S3 Appendix — Magnetic field maps which have a dipolar-like pattern can also be transformed by solving the source reconstruction with an ECD fit. In this appendix, we show the methods and results of transforming AEF measurements and simulations. (PDF) [file pone.0262669.s003.pdf]

# **S3 Appendix**. Transforming MEG data using an equivalent current dipole fit approach.

In the main paper we have shown, that we can successfully transform data of the same measured phenomenon between two magnetoencephalography (MEG) systems using the minimum norm estimate (MNE) algorithm. We used two different implementations of MNE, one uses the spherical volume conductor (MNE-SPH) and the other uses an individual geometry of each subject (MNE-BEM). In this appendix, we show that for a special case, where we have a focal brain activity, like auditory evoked fields (AEF), we can transform data between two MEG systems using an equivalent current dipole (ECD) fit. The key idea is the same as for the transformation methods in the main paper, we calculate the source reconstruction at one specific time. Using the inverse solution of one and the forward model of the other system, we can calculate the magnetic fields on the other system. Here we used two different methods: fitting a dipole implemented in the software package MNE-Python, which uses a forward model of a source inside a 3 layer shell conductor, it is solved using the boundary element method (BEM) (ECD-BEM); simplified implementation of dipole fitting, using the forward model of source inside a spherical volume conductor (ECD-SPH). The sketch of both methods is shown in Fig 1. Here we present the results of transforming both measurements and simulations of AEFs, which were also used in the main paper. We used only the right half of the SQUID sensors, since the OPM sensors measured exclusively in the vicinity of the right auditory cortex.

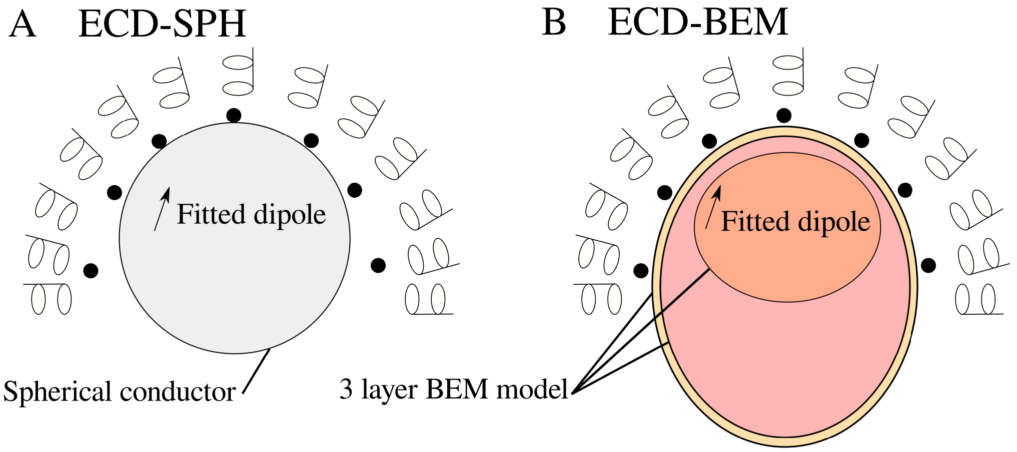


**Fig 1. Schematic representation of two different transformation techniques.** (A) ECD fitted inside a homogeneous spherical volume conductor model (ECD-SPH method); (B) fitted ECD using individual geometry of the head (ECD-BEM method). On the figures, sensors from both MEG systems are drawn, we did not use them simultaneously.

### **Dipole fit inside a homogeneous spherical volume conductor (ECD-SPH).**

This method fits one equivalent current dipole (ECD). The forward solution is calculated with the simplified formula of the source inside a homogeneous spherical

volume conductor [1]. The exact formula for which we calculated the magnetic field is written in the first paragraph of supplementary material **S2 Appendix**. With this model, the magnetic field can be calculated analytically without the use of the subject's individual geometry (BEM). The sensor model in this implementation did not take into account the real sensor geometry, same as in MNE-SPH. The sphere, which represents the volume conductor, was fitted so that it best fits the outer brain surface. The inverse problem is solved by performing a nonlinear least-squares curve fitting of $\vec{p}$ (ECD direction) and $\vec{r}_p$ (ECD location) using the Levenberg-Marquardt algorithm [2]. For this algorithm we used a implementation (function "leastsq") in python package SciPy [3]. It is well known that this method can find a local minimum instead of a global one, therefore it is necessary to start fitting with a good initial guess of the source parameters.

To minimize this problem we first perform a linear fit to estimate only the direction and strength of the initial dipole for a set of fixed locations [4]. For one fixed location $\vec{r}_p$ we calculated the lead field $\vec{L}$, same as in the third paragraph of supplementary material (**S2 Appendix**). To obtain the $\vec{p}$, we solved the following equation:

$$\vec{p} = (\mathbf{L}^{\mathrm{T}}\mathbf{L})^{-1}\mathbf{L}^{\mathrm{T}}\mathbf{B} \tag{1}$$

where $\mathbf{L} = (\vec{L}_1, \ldots, \vec{L}_n)$, $n$ is the total number of sensors and $\mathbf{B} = (B_1, B_2, \ldots, B_n)$ the magnetic field map. To solve the inverse $(\mathbf{L}^{\mathrm{T}}\mathbf{L})^{-1}$ we used the Moore-Penrose pseudo-inverse method implemented in Python software package NumPy [5]. The location, direction and strength ($\vec{r}_p$, $\vec{p}$) with the lowest RE between the original and reconstructed data is chosen. We apply these results as the starting parameters in the nonlinear fitting procedure to obtain the final parameters of the dipole (location, direction, and strength of the ECD). To calculate the magnetic field on the other MEG measuring system, we have to first transform the ECD coordinates and direction into the coordinate system of the other MEG system, then we can calculate the magnetic field on the measuring sites of the other system using the formula for magnetic field inside a spherical volume conductor (first paragraph of **S2 Appendix**).

### Dipole fit implemented in MNE-Python using subject's individual BEM model (ECD-BEM).

[6] This algorithm fits one dipole for each measured magnetic field map (MFM) and is conceptually similar to the ECD-SPH method. This method uses the core functions implemented in the software package MNE-Python, all details are accessible in [6, 7] and can also be found in the documentation of the software package MNE Python on its web page https://mne.tools/stable/overview/index.html. The magnetic fields are calculated using the individual subject's head geometry (BEM model), the detailed formula is presented in the second paragraph of supplementary material (**S2 Appendix**). The calculation takes into account the noise covariance matrix, i.e., individual channels have different weights. The sensor model in this implementation takes into account the real sensor geometry, same as MNE-BEM.

An example of commands that we used in our code to transform the data from one system to another and vice versa are shown on Fig 2. First, we import the averaged data, which we previously preprocessed, we crop it to have only the data of the M100 peak, which we want to transform. Next, we perform the dipole fit using the noise covariance matrix, which we calculated during the preprocessing, and the BEM model. For previously fitted dipole, we create a forward model and source estimate on the other system. Here we have to be careful with the coordinate systems (transformations), to use the code in Fig 2, both MEG systems have to be in the same coordinate system. Lastly, we can calculate the magnetic field with the forward operator and the source estimate.

```
import mne

# import the averaged Evoked data object
squid_evoked = mne.Evoked(evoked_squid_path)
opm_evoked = mne.Evoked(evoked_opm_path)

# get only the data of M100 (peak_opm is the M100 time for the OPM MEG system)
opm_evoked = opm_evoked.crop(peak_opm, peak_opm)
squid_evoked = squid_evoked.crop(peak_squid, peak_squid)

# compute the forward operator, for the opms we need a sensor definition file (coil_def_
# fname), fname_trans is the transformation file from head to MRI, src is the source space,
# bem_sol is the BEM solution
with mne.use_coil_def(coil_def_fname):
    fwd_opm = mne.make_forward_solution(opm_evoked.info, fname_trans_opm, src, bem_sol,
                                        ignore_ref=True)
fwd_squid = mne.make_forward_solution(squid_evoked.info, fname_trans_squid, src, bem_sol,
                                      ignore_ref=True)

# read the noise covariance matrix, which was calculated during the preprocessing step
noise_cov_opm = mne.read_cov(noise_covariance_opm_path)
noise_cov_squid = mne.read_cov(noise_covariance_squid_path)

# compute the inverse operator, the parameters depth, loose and fixed can be changed
inv_squid = mne.minimum_norm.make_inverse_operator(squid_evoked.info, fwd_squid,
                                        noise_cov_squid, depth=None, loose=0, fixed=True)
inv_opm = mne.minimum_norm.make_inverse_operator(opm_evoked.info, fwd_opm,
                                        noise_cov_opm, depth=None, loose=0, fixed=True)

# apply the inverse operator and calculate the the source estimates
stc_squid = mne.minimum_norm.apply_inverse(squid_evoked, inv_squid, lambda2=1/9,
                                           method="MNE", pick_ori=None)
stc_opm = mne.minimum_norm.apply_inverse(opm_evoked, inv_opm, lambda2=1/9,
                                         method="MNE", pick_ori=None)

# calculate the magnetic fields on the other system
transformed_opm_evoked = mne.apply_forward(fwd_opm, stc_squid, opm_evoked.info)
transformed_squid_evoked = mne.apply_forward(fwd_squid, stc_opm, squid_evoked.info)
```

**Fig 2. Software code of the ECD-BEM method.** On this figure are all the crucial functions to transform measurements from one MEG system to the other using the ECD fit implemented in MNE-Python.

# Results and Discussion

The results of transforming measured data for both techniques (ECD-SPH and ECD-BEM) are shown in Fig 3. The left subplot shows the averaged value of relative errors (REs) and the right subplot the averaged value of correlation coefficients (CCs) for all subjects when comparing the MFMs at the time of the M100 peak. RE and CC were calculated for four cases: when we compared the measured SQUID data and the measured OPM transformed to SQUID sensors (in the figure labeled as SQUID); when we compared the measured OPM data and the measured SQUID data transformed to OPM sensors (in the figure labeled as OPM); when we compared the measured and reconstructed SQUID data and when we compared the measured and reconstructed OPM data. From Fig 3, one can quickly see that the transformation with ECD-BEM performs better than ECD-SPH (higher RE and lower CC). However, this is not the case for the reconstruction errors, results are very similar.

Next, we show the results of both transformation methods of simulated data for different noise levels (different signal-to-noise ratio (SNR)) in Fig 4: (A) ECD-SPH; (B)

ECD-BEM. The figures of all subplots are the result of averaging 10 times for different added random noise. For high and low values of SNR (low and high noise levels). Both methods perform well. For low strengths of noise (high SNR), the methods ECD-BEM has slightly lower errors (transformation and reconstruction) because we fit the ECD with the same forward model as the simulations were performed.

Generally, the MNE-Python implementation of the ECD provides better results, which is expected since the AEF originated from a focal source and the realistic head model is considered. ECD-BEM and ECD-SPH can be applied only, when one hemispheric data is considered or when one focal source is expected. Results show, that for a special case of focal sources, also an ECD fit can be used for data transformation, however, the MNE source model is preferred in general use.

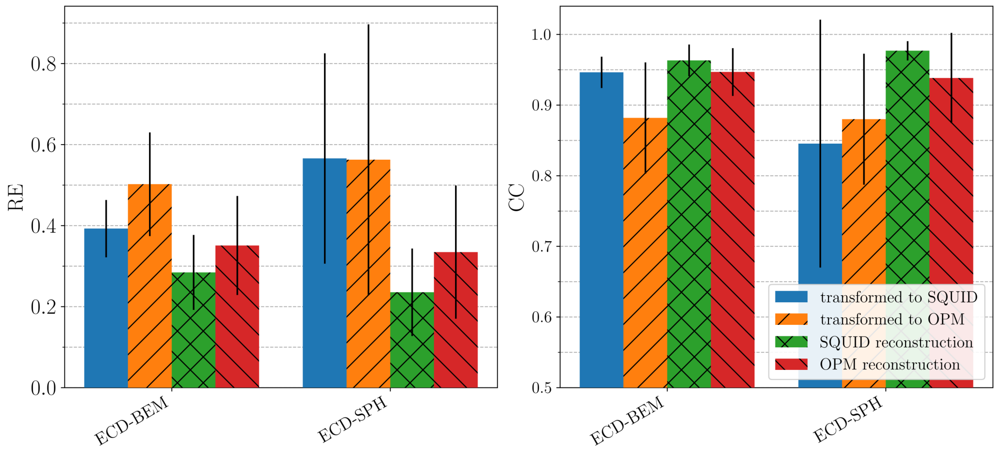


**Fig 3. Comparison of different transformation methods for the measurements.** The left image displays the relative error (RE) and the right the correlation coefficient (CC) between the measured original and the transformed magnetic field map from the other system. The orange column shows the results of the transformation of the SQUID data to the OPM-MEG system and the blue column vice versa. The green and red columns show the REs and CCs for comparison between original and reconstructed data. The black lines on top of the bars represent the standard deviation of REs and CCs.

## References


1. Sarvas J. Basic mathematical and electromagnetic concepts of the biomagnetic inverse problem. Physics in Medicine and Biology. 1987;32(1):11–22. doi:10.1088/0031-9155/32/1/004.

2. Press WH, editor. FORTRAN numerical recipes. 2nd ed. Cambridge [England] ; New York: Cambridge University Press; 1996.

3. SciPy 1 0 Contributors, Virtanen P, Gommers R, Oliphant TE, Haberland M, Reddy T, et al. SciPy 1.0: fundamental algorithms for scientific computing in Python. Nature Methods. 2020;17(3):261–272. doi:10.1038/s41592-019-0686-2.

4. Ilmoniemi R, Sarvas J. Brain signals: physics and mathematics of MEG and EEG. Cambridge, Massachusetts: The MIT Press; 2019.

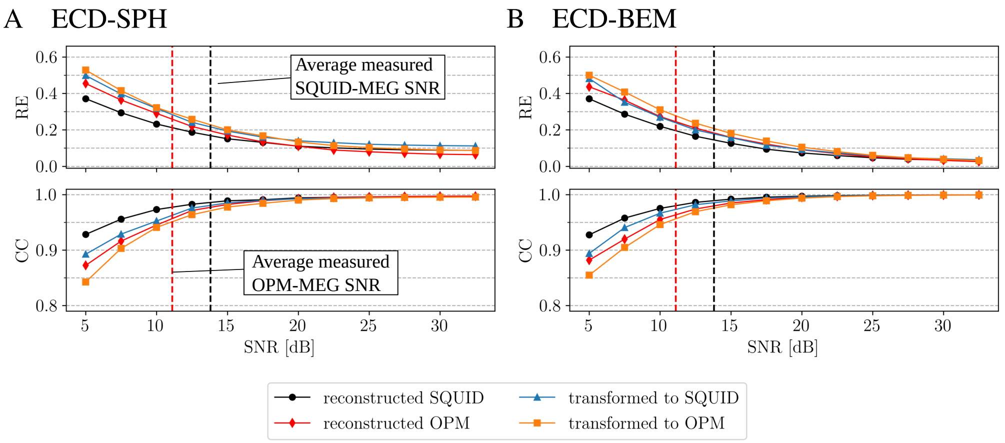


**Fig 4. Transformation of simulated data using the method as indicated.** Each subfigure shows the averaged RE and CC vs. SNR for four cases: comparison of simulated and reconstructed SQUID data; comparison of simulated and reconstructed OPM data; comparison of simulated SQUID data and transformed simulated OPM data to SQUID system; comparison of simulated OPM data and transformed simulated SQUID data to OPM system. Each subfigure is the result of averaging 10 times for different random added noise. The dashed vertical lines show average values of SNR, which were calculated from the measured data for each MEG system.

5. Harris CR, Millman KJ, van der Walt SJ, Gommers R, Virtanen P, Cournapeau D, et al. Array Programming with NumPy. Nature. 2020;585(7825):357–362. doi:10.1038/s41586-020-2649-2.

6. Gramfort A. MEG and EEG data analysis with MNE-Python. Frontiers in Neuroscience. 2013;7. doi:10.3389/fnins.2013.00267.

7. Gramfort A, Luessi M, Larson E, Engemann DA, Strohmeier D, Brodbeck C, et al. MNE software for processing MEG and EEG data. NeuroImage. 2014;86:446–460. doi:10.1016/j.neuroimage.2013.10.027.
