# Supplementary material for: Transforming and comparing data between standard SQUID and OPM-MEG systems
Source: PLoS One. 2022 Jan 19;17(1):e0262669. doi: 10.1371/journal.pone.0262669 (PMC8769297; doi:10.1371/journal.pone.0262669)
Supplement: S1 Table — This table lists all the acronyms and abbreviations that appear in the text at least three times with full names. (PDF) [file pone.0262669.s006.pdf]

S1 Table. Acronyms and abbreviations.

Acronym	Full name
MEG	magnetoencephalography
SQUID	superconducting quantum interference device
OPM	optically pumped magnetometer
AEF	auditory evoked fields
MRI	magnetic resonance imaging
BEM	boundary element method
MNE	minimum norm estimate
RE	relative error
CC	correlation coefficient
MFM	magnetic field map
MSR	magnetically shielded room
SNR	signal-to-noise ratio
STD	standard deviation
ECD	equivalent current dipole
ANOVA	analysis of variance
RMS	root mean square
CAD	computer-aided design
PTB	Physikalisch-Technische Bundesanstalt
LSD	least significant difference

This table lists all the acronyms and abbreviations with full names, which appear in the text at least three times.
